# Toward Rapid Actinium-225 Purification via Membrane Adsorbers with Covalently Tethered Diglycolamide Ligands

**DOI:** 10.1021/acsami.5c17147

**Published:** 2026-02-05

**Authors:** Shruti Krishna Radhakrishnan, Megan M. Sibley, Bernadette L. Schneider, Pavithra H. A. Kankanamalage, Tuli Banik, Tae Kyong John Kim, Jasmine Hatcher-Lamarre, Luke A.F. Venturina, Timothy Yen, Joshua T. Damron, Alec Johnson, Alexa G. Ford, Trent Kozar, Tugce Uz, Weimin Zhou, Cathy S. Cutler, Christine E. Duval

**Affiliations:** † Department of Chemical & Biomolecular Engineering, 2546 Case Western Reserve University, Cleveland, Ohio 44106, United States; ‡ Isotope Research and Production Department, 8099 Brookhaven National Laboratory, Upton, New York 11973, United States; § Chemical Sciences Division, 6146 Oak Ridge National Laboratory, Oak Ridge, Tennessee 37831, United States; ∥ Swagelok Center for Surface Analysis of Materials, 2546 Case Western Reserve University, Cleveland, Ohio 44106, United States

**Keywords:** extraction chromatography, membrane adsorbers, diglycolamides, ^225^Ac, targeted alpha therapy

## Abstract

Extractive diglycolamide (DGA) resins are used in several state-of-the-art techniques for purifying ^225^Ac, a promising radiometal for targeted alpha therapy. Unfortunately, separation processes that rely on resins are often limited to slow flow rates, high elution volumes, and long processing times. Membrane adsorbers functionalized with DGA ligands are an alternative separation material that may overcome these challenges. This work presents (1) the synthesis of an aminated tetrahexyldiglycolamide ligand, (2) the covalent tethering of the ligand to electrospun poly­(vinylbenzyl chloride) fiber mats, and (3) the adsorption and desorption of La­(III) and ^225^Ac. Chemical and physical characterization supports the covalent tethering of the ligand to the fiber mat, as well as the preservation of the fiber surface area and porosity after functionalization. Equilibrium adsorption experiments were performed with stable La­(III) and radioactive ^225^Ac. Trends in affinity are consistent between commercial resins and the synthesized membrane adsorbers; however, the Langmuir constants and the maximum binding capacity of the membrane adsorbers were generally lower than the resins. Despite these differences, the modeled selectivity for an equimolar solution of La­(III)/^225^Ac in 10 M nitric acid is 57. Furthermore, ^225^Ac is rapidly desorbed from the fibers in 10 M nitric acid (<20 min). The La­(III)/^225^Ac selectivity and rapid ^225^Ac desorption indicate this class of materials is promising for rapid radioanalytical separations.

## Graphical abstract

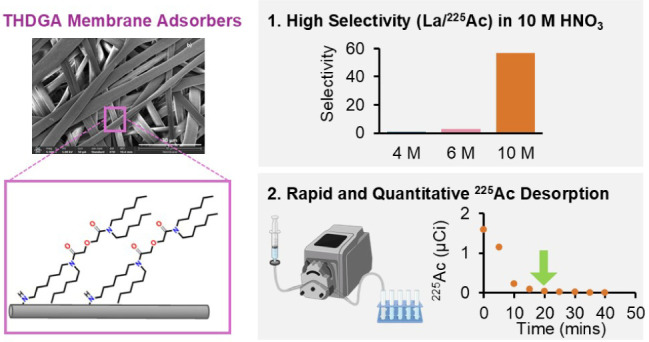

## 1 Introduction

^225^Ac conjugates are emerging radiopharmaceuticals used to treat cancer via targeted alpha therapy.^1^ To date, ^225^Ac has been sourced by three primary methods: (1) isolation of the parent isotope, ^229^Th, from legacy ^233^U waste^2^ for use in a generator, (2) spallation of thorium targets in a particle accelerator,^3–6^ and (3) irradiation of thorium targets via proton- or photon-induced reactions.^7–9^ The global production of ^225^Ac from legacy waste is approximately 1.7 Ci per year^2^ and the implementation of spallation production routes is increasing that amount.^10^ Before conjugation to the cancer-targeting biomolecule, ^225^Ac must be isolated from other radiometals (Th, Ra, and/or lanthanides) that may interfere with the conjugation chemistry.

The current state-of-the-art method for purifying clinical ^225^Ac, regardless of production method, is to use a series of resin-packed columns. Oak Ridge National Laboratory developed a process that relies entirely on AG50 × 4 cation exchange resin (Bio-Rad) and AG MP-1 anion exchange resin (Bio-Rad) to achieve the final ^225^Ac product.^11,12^ Recently, TerraPower Isotopes developed a purification process that uses AG MP-1M anion exchange (Bio-Rad) and UTEVA (Eichrom Inc.) to remove the ^229^Th. Then, the final purification of ^225^Ac is achieved using an extractive resin, branched DGA (Eichrom Inc.).^2^ Los Alamos National Laboratory developed a two-column process in which the dissolved Th target is converted to a Th-citrate complex which is separated from the spallation products using an anion exchange column. Then, ^225^Ac is isolated from the remaining divalent and trivalent spallation products using branched DGA resin through multiple washing steps.^13,14^ A commonality between the emerging separation processes is the final step in which ^225^Ac is selectively purified using a branched DGA resin-packed column and concentrated acid (4–10 M nitric acid).^2,13,14^

Branched DGA resins are synthesized by physically absorbing tetra-2-ethylhexyl-diglycolamide (TEHDGA) ligands into a mesoporous (25 nm pore diameter) acrylic resin^15,16^ which presents two possible challenges. First, resin-packed columns are known to exhibit diffusion-limited transport^17^ which results in slow operational flow rates to achieve high product capture.^18^ Practically, this means the final elution step for ^225^Ac purification can last >6 h at flow rates of 0.2 mL/min. Second, physisorbed ligands are prone to leaching from the resins which may add additional cleanup steps prior to conjugation with the drug.

An alternative to resins is membrane adsorbers with micropores (0.1–1 μm diameter) in which adsorbate transport to the binding sites is driven by convection instead of diffusion. Overcoming the mass transport limitations imposed by resins opens the door for high productivity separations where product capture is not a function of flow rate.^18,19^ To transition from mesoporous to microporous materials, it is necessary to covalently tether the functional ligands to the substrates. Furthermore, covalently bound ligands may limit ligand leaching at high flow rates.

Diglycolamide ligands and their derivatives have been covalently tethered to a variety of chromatography media like mesoporous silica,^20–24^ mesoporous carbon,^25^ and polymer resins.^26,27^ The adsorption of trivalent lanthanides and actinides on these materials has been characterized using several common metrics like the distribution coefficient (*K*_d_ or *D*_w_), Langmuir association constant, and maximum binding capacity. Bertelsen et al. emphasized the importance of characterizing new extractive materials using common language or metrics for comparison with the broader extractive resin literature.^28^ Florek et al. emphasized that new separation materials must be characterized with sufficient detail to provide useful information for material development.^20^ Often, newly synthesized materials for radioanalytical chemistry are only evaluated for the distribution coefficient (*K*_d_) which is measured when the ligand is in 50x excess of the metal.^15^ Thus, a *K*_d_ will only be valid for dilute solutions and does not capture concentration-dependent effects of binding. Florek et al. recommend (1) measuring the full equilibrium adsorption isotherm; (2) performing kinetic studies; (3) assessing stability over multiple load/elution cycles and (4) conducting dynamic (flow-through) separation experiments.^20^

In this work, membrane adsorbers were synthesized by covalently tethering tetraethylhexyl diglycolamide (THDGA) ligands to electrospun poly­(vinylbenzyl chloride) (PVBC) membranes. Lanthanum (La­(III)) was chosen as a nonradioactive surrogate for the comparative study of THDGA membranes and commercial linear DGA (TODGA) resins. Despite known differences in the adsorption of La­(III) and ^225^Ac to the commercial TODGA resins at high acid concentration (>8 M nitric acid),^15^ La­(III) has a comparable charge and ionic character to ^225^Ac.^29^ It served the dual purpose of a nonradioactive surrogate to prepare for ^225^Ac experiments and as a representative lanthanide to directly compare THDGA membranes to TODGA resins. We take the first steps toward full characterization by measuring full equilibrium adsorption isotherms and conducting dynamic (flow-through) experiments to load and elute ^225^Ac from the THDGA membranes. While this initial study compares the adsorption and elution of La­(III) and ^225^Ac, we anticipate that they will be useful for other medically important radionuclides like ^177^Lu and ^47^Sc.^30–32^ Future work will continue the characterization of the materials to understand the full impact of the design.

## 2 Materials

All chemicals were purchased from commercial sources and used as received unless otherwise specified. Poly­(vinylbenzyl chloride) (PVBC) was purchased from Scientific Polymer Products (60/40 mixture of *m*- and *p*- isomers, MW 500 000 g/mol). Tetrahydrofuran (THF) (unstabilized, HPLC grade, OmniSolve, Millipore Sigma) and lithium chloride (LiCl, 99+%, Acros Organics) were used for electrospinning membranes.

Solvents used in the ligand synthesis, column chromatography, chemical reaction of ligand to membrane, and characterization were tetrahydrofuran (THF) (stabilized with ∼0.025% butylated hydroxytoluene, certified, Fisher), dichloromethane (DCM) (stabilized; Laboratory Plus, Honeywell), ethyl acetate (EtOAc) (certified ACS, Fisher), chloroform (CHCl_3_) (stabilized with 2-methyl-2-butene, 99.5% HPLC grade, TCI America) methanol (MeOH) (≥99.8% certified ACS, Fisher), and ethanol (EtOH) (Absolute 200 proof, 99.5+% certified ACS, Acros Organics). If solvents are indicated as dry, they were obtained by storage over 3 Å molecular sieves (1–2 mm diameter pellets, Thermo Scientific). Deionized (DI) water (10 MΩ) was obtained from a RiOS 3 water purification system (Millipore Sigma, Burlington, Massachusetts).

The diglycolamide-type ligand was synthesized using 1,6-hexanediamine (99.5%, Acros), hexyl bromide (>98.0%, TCI America), ethyl trifluoroacetate (99.8%, Chem Impex International), diglycolic acid (98%, Acros Organics), thionyl chloride (1M solution in dichloromethane, TCI America), triethylamine (99% HPLC grade, Fisher), and potassium hydroxide (85% technical grade flakes, Fisher). Silica gel (SiliaFlash 40–63 μm, 60 Å, Silicycle) was used for column chromatography. Aluminum-backed silica plates (Silicycle, SiliaPlate, 200 μm, Indicator F-254) were used for thin layer chromatography. Potassium iodide (KI) (certified ACS, Fisher) and cesium carbonate (Cs_2_CO_3_) (99%, Alfa Aesar) were used in reaction of PVBC membranes with the synthesized aminated-diglycolamide ligand.

Deuterated solvents for NMR were chloroform-d (CDCl_3_) (99.8% + 0.03% v/v tetramethylsilane (TMS), Cambridge Isotope Lab), which was initially used as received and later stored over 4 Å molecular sieves (8–12 mesh beads, Fisher) or dimethyl sulfoxide-*d*_6_ (DMSO-*d*_6_) (99.5%, Alfa Aesar).

La­(III) adsorption experiments at Case Western Reserve University used concentrated nitric acid (Fisher Optima grade, 67–70% for Ultra Trace Elemental Analysis), lanthanum in nitric acid purchased from High Purity Standards (1000 ± 3 μg/mL lanthanum from lanthanum oxide, 2% HNO_3_), and deionized water (DI water) from a RIOS 3 water purification system. Commercial TODGA resins were purchased from Eichrom (TODGA resin, Normal, 50–100 μm) which uses the N,N,N′,*N*′-tetra-n-octyldiglycolamide (TODGA) ligand as an extractant.

^225^Ac was sourced from Brookhaven National Laboratory. ^225^Ac adsorption experiments at Brookhaven National Lab used concentrated nitric acid (Fisher Optima grade, 67–70%) and ultrapure water (18.2 MΩ) from a Milli-Q IQ 7000 Ultrapure Lab Water System.

## 3 Methods

### 3.1 Synthesis of aTHDGA

The aminated tetrahexyldiglycolamide (aTHDGA) ligand synthesis is summarized in Figure 1. All reagents and solvents were of reagent quality and were used as received, listed in Section 2. All syntheses and purifications were performed at ambient conditions unless otherwise specified. The Supporting Information contains detailed synthetic information for the intermediate and final molecules as well as identified chemical shifts δ (ppm) and coupling constants *J* (Hz) from the NMR spectra.

**Figure 1:**
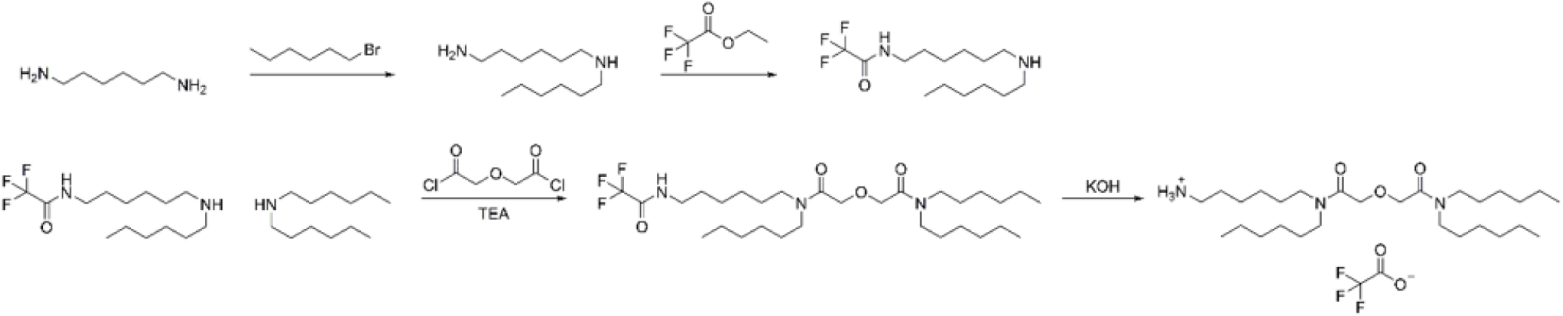
aTHDGA ligand synthesis.

### 3.2 ^1^H,^13^C,^19^F Nuclear Magnetic Resonance Spectroscopy (NMR)

Solution-phase ^1^H, ^13^C, and ^19^F NMR spectra were recorded using a Bruker Ascend 500 MHz spectrometer with Prodigy liquid nitrogen cryoprobe. Chemical shifts δ (in ppm) are referenced to tetramethylsilane using the residual solvent as an internal standard for ^1^H and ^13^C. Coupling constants (*J*) are expressed in hertz (Hz).

### 3.3 High-Resolution Mass Spectrometry

High-resolution mass spectrometry (HRMS) and liquid chromatography–mass spectrometry (LC-MS) were performed at Auburn University Mass Spectrometry Center on a quadrupole Orbitrap Exploris 120 mass spectrometer with electrospray ionization in positive mode using Xcalibur software (V4.4.16.14). LC-MS used a Vanquish Binary UHPLC, Waters Acquity UPLC BEH C18 column (130 Å, 1.7 μm, 2.1 mm × 50 mm) prior to mass spectrometry, with mobile phase solution A (95% water 5% acetonitrile with 0.1% formic acid) and linear ramp to 100% solution B (95% acetonitrile 5% water with 0.1% formic acid).

### 3.4 Formation and Functionalization of PVBC Membranes with aTHDGA

PVBC membranes were synthesized using a home-built electrospinning system.^33^ The polymer dope was prepared by dissolving PVBC (MW 500 000) in tetrahydrofuran with lithium chloride. The final composition of the dope solution was 15 wt % PVBC and 0.5 wt % LiCl. Electrospinning was performed at a flow rate of 7 mL/h, voltage of 20 kV, relative humidity of 40–50%, and needle-to-plate distance of 8 cm. Fibers were spun onto filter paper (P4, Fisher Scientific) for both ease of handling and for aiding in the wettability in the reaction with aTHDGA. Membranes were cut using a 44 mm die-punch for the functionalization reactions.

PVBC membranes were functionalized with aTHDGA according to the reaction shown in Figure 2. The detailed synthesis is described in the Supporting Information. Briefly, membranes were submerged in an ethanol solution of aTHDGA with cesium carbonate and potassium iodide then heated to reflux. The reaction covalently bonds the aTHDGA to the surface of the fibers via nucleophilic substitution. The reacted membranes (THDGA membranes) were then washed with water and ethanol to remove unbound aTHDGA.

### 3.5 Scanning Electron Microscopy (SEM)

Membrane and resin morphologies were examined using a Thermo Fisher Apreo 2 scanning electron microscope (SEM). The Everhart–Thornley detector was employed for imaging THDGA membranes and PVBC membranes, while the T2 detector was used for TODGA resins. Sample preparation for SEM involved mounting the membranes and resins on SEM pin stubs with double-sided carbon tape, followed by sputter-coating with palladium using a Denton Vacuum DESK V Sputter Coater for 60 s. Imaging was conducted at a working distance of 10 mm, with a current of 50 pA, an accelerating electron voltage of 5.00 kV, and a tilt angle of 0°. The magnification ranged from 1,000x to 120,000x for resin samples and 1,000x to 10,000x for membranes. Fiber diameters of both THDGA and PVBC membranes, as well as particle diameters of the TODGA resins, were measured using the Apreo 2S software.

### 3.6 X-ray Photoelectron Spectroscopy (XPS)

X-ray photoelectron spectroscopy (XPS) was performed using a PHI 5000 Versaprobe XPS instrument with a monochromatized Al Kα source. For membranes, pieces (<3 mm^2^) of membrane were mounted on a 1-inch holder with carbon tape (Ted Pella, 5 mm width). For the aTHDGA ligand oil, ligand was dissolved in minimal chloroform, dried with 3 Å molecular sieves in a sealed vial overnight, then dropcast onto a clean silicon wafer (1 cm^2^) and placed under vacuum to dry overnight (ambient temperature, 27 in Hg vacuum). The wafer was mounted onto a 1-inch holder placed under a heat lamp for about 10 min to ensure solvent was thoroughly evaporated before introducing the sample into the vacuum chamber. Total pressure in the vacuum chamber was in the range of 1 × 10^–6^ to 1 × 10^–7^ Pa.

### 3.7 Time of Flight–Secondary Ion Mass Spectrometry (ToF-SIMS)

ToF-SIMS analyses were performed using a TRIFT V nanoTOF (Physical Electronics) instrument. The sample surface was cleaned using 3 kV Ar gas gun before measurement to remove surface contamination. The analytical chamber had a pressure of ∼2 × 10^–9^ Torr during the analysis. Thirty keV Ga^+^ pulsed primary ion source was used for all measurements (bunched mode, positive polarity, scan size 200 μm × 200 μm). Charge compensation was achieved in dual mode using electron gun and gas gun. Mass spectra were collected in the mass-to-charge range of 0–1850 *m*/*z*. The data were analyzed using WincadenceN 1.8.1 software program from Physical Electronics.

### 3.8 Attenuated Total Reflectance Fourier Transform Infrared Spectroscopy (ATR-FTIR)

Fourier-transform infrared spectroscopy (FTIR) was performed using the attenuated total reflectance (ATR) diamond crystal accessory of a Nicolet iS50 FTIR instrument (Thermo Scientific). Each measurement was collected as 32 scans with a resolution of 2 cm^–1^ with background subtraction. Baseline correction and spectra normalization were performed with Omnic 9 software, version 9.11.727. The ligand was prepared by dissolving in minimal CHCl_3_ and drying over 3 Å molecular sieves in a sealed vial overnight to remove any absorbed water. The solution was transferred to a clean glass coverslip and the solvent was evaporated with gentle heating from a heat gun applied to the reverse side of the glass. The dried viscous oil was then transferred onto the ATR diamond crystal for analysis.

### 3.9 Solid-State NMR

Cross polarized total sideband suppression (CP-TOSS) spectra were acquired at ambient temperature (∼25 °C) on a Bruker AVANCE III 400 MHz spectrometer with Bruker 3.2 mm triple-resonance MAS probe. The samples were packed separately into zirconia rotors and were spun at 12 kHz.

### 3.10 Membrane Porosity

The porosity of THDGA membranes and PVBC membranes was determined by cutting the membranes using a die-cutter or trimming them to a similar mass (0.0018 ± 0.0002 mg) after scraping them from the filter paper backing. The membranes were then soaked in isopropanol, a nonswelling solvent, on a shaker table at 75 rpm for 24 h. The porosity was subsequently calculated using eq 1.

1 $$\varepsilon =\frac{\frac{m_{w}-m_{d}}{\rho_{i}}}{\frac{m_{w}-m_{d}}{\rho_{i}}+\frac{m_{d}}{\rho_{p}}}$$

Where *m*_w_ and *m*_d_ represent the masses (g) of the wet and dry membranes, respectively, and ρ_*i*_ and ρ_p_ denote the densities of isopropanol and the polymer (g/cm^3^), respectively. The polymer density was estimated to be 1.088 g/cm^3^ based on the manufacturer’s specifications.

### 3.11 Membrane Permeance

The pure water flux (*J*_w_) of THDGA and PVBC membranes was used to calculate the membrane permeance, *A* (LMH/bar). Flux was measured by pumping deionized (DI) water through each membrane situation within a syringe filter holder (PALL 4320 25 mm Easy Pressure Syringe Filter Holder). The feed was pressurized in a 10 L stainless steel tank using a compressed air cylinder (UN1002, Airgas) at three different pressures: 10, 15, and 20 psi (0.69–1.38 bar). The permeate was collected and weighed using a digital balance (OHAUS Ranger7000, OHAUS, New Jersey). Three replicate measurements were performed on a single membrane at each pressure. Pure water flux data were plotted against transmembrane pressure to calculate the membrane permeance, where the slope of the resulting straight lines represented the permeance (*A*).

### 3.12 THDGA Membrane and Resin Equilibrium Adsorption: La­(III)

The maximum capacity and the affinity of TODGA resins and THDGA membranes were determined through equilibrium adsorption experiments. First, 2.16 × 10^–3^ – 2.16 mM La­(III) solutions were each prepared in 4, 6, and 10 M nitric acid. Next, 10.0 ± 0.3 mg of resin or membrane were weighed and added to a 2 mL microcentrifuge tube. Then, 1 mL of La­(III)-containing solution was added to each centrifuge tube using a calibrated micropipette. Finally, adsorbents were equilibrated for 24 h in a rotisserie tube rotator at an ambient temperature. After equilibration, all samples were filtered using PTFE syringe filters (VWR International, LLC) before analysis. The samples were then diluted using DI water to 2–5 wt % nitric acid for analysis by inductively coupled plasma optical emission spectroscopy (ICP-OES, Agilent Technologies, Santa Clara, CA, USA). The calibration standards were prepared by serial dilutions of 7.2 mM La­(III) OES standard in 2 wt % nitric acid (High Purity Standards). The intensity of the emission wavelength of La­(III) at 379.477 nm was used for all the calculations. The equilibrium adsorption capacity was calculated by a mass balance, shown in eq 2, and the maximum adsorption capacity was calculated using the Langmuir isotherm, eq 3.

2 $$Q_{e}=\frac{\left(C_{o}-C_{\mathrm{eq}}\right)}{m}\times V$$

3 $$Q_{e}=\frac{Q_{\max}\times K_{L}\times C_{\mathrm{eq}}}{1+K_{L}\times C_{\mathrm{eq}}}$$

Where *Q*_e_ is the equilibrium adsorption capacity (mmol La­(III)/g of adsorbent), *V* is the volume of the solution (L), *m* is the mass of adsorbent (g), *K*_L_ is the Langmuir constant (mM^–1^) and *C*_o_ and *C*_e_ are the initial and equilibrium concentrations of La­(III) (mM), respectively. The Langmuir isotherm was used to model the adsorption data, and the model-fitting parameters were obtained by nonlinear regression in Origin 2023 software using the Levenberg–Marquardt technique.

### 3.13 THDGA Membrane Desorption Experiments: La­(III)

THDGA membranes (∼10 mg) loaded with La­(III) from the equilibrium adsorption experiments were mounted in a syringe filter membrane housing (PALL 4320 25 mm Easy Pressure Syringe Filter Holder). A pH 2 nitric acid eluent was delivered to the membrane housing using a peristaltic pump (MasterFlex) at volumetric flow rate of 0.5 mL/min. During the experiment, 2 mL fractions were collected continuously using an automated fraction collector (Bio-Rad) for 1 h. The La­(III) concentration in the effluent fractions was quantified by ICP-OES according to the procedures described in Section 3.12.

### 3.14 THDGA Membrane Equilibrium Adsorption Experiments: ^225^Ac

Nitric acid solutions of 4, 6, and 10 M were prepared by diluting concentrated nitric acid (Fisher Optima grade, 67–70%) with ultrapure water. ^225^Ac was supplied dissolved in 125 μL of 0.1 M hydrochloric acid and was diluted with 875 μL of 4 M nitric acid. This ^225^Ac stock solution was further diluted to 7.4–11.4 μCi/mL with 4, 6, or 10 M nitric acid.

^225^Ac adsorption experiments using THDGA membranes were performed below the maximum binding capacity of the membranes. THDGA membranes with masses of 0.25, 0.50, 0.75, 1.00, and 1.25 mg were measured using an analytical microbalance (C31 microbalance, Cahn Instruments) at Cleveland State University and placed inside 2 mL polypropylene centrifuge tubes (Fisherbrand). For each experiment, 1.000 mL of the ^225^Ac containing solution was pipetted into a THDGA membrane-containing centrifuge tube, and the sample was analyzed via gamma spectroscopy (vide infre) to quantify the initial radioactivity of ^225^Ac. Then, the THDGA membrane was equilibrated with the ^225^Ac solutions for 3 h on a shaker table (ThermoFisher Scientific Compact Digital Rocker) at 100 rpm. After 3 h, the membrane was separated from the supernatant via centrifugation (Bio-Rad Model 16K microcentrifuge) at 12000 rpm for 10 min. A 0.700 mL aliquot of supernatant was decanted into a 2 mL microcentrifuge tube and reconstituted with 0.300 mL of water for quantification. Control samples without THDGA membranes were performed similarly.

^225^Ac quantification was performed by directly measuring the gamma emission of daughter product, ^221^Fr (218.12 keV) using an Ortec Gem30P4–83 High Purity Germanium (HPGe) coaxial detector. The HPGe gamma spectroscopy instrument used a Mobius-PT-DET liquid nitrogen recycler dewar and CFG-PV4 cryostat configuration with the GammaVision software (version 8.10.02, Advanced Measurement Technology, Inc.). The gamma spectroscopy measurements were performed at least 20 h after completion of the adsorption or desorption experiments, to allow sufficient time for the ^225^Ac to establish secular equilibrium with its decay products.^34^ Gamma spectra were collected with 1000–3000 s of live time. Decay corrections were applied to calculate the activity of ^225^Ac at the time of the experiment in the original 1 mL volume. The radioactivity of ^225^Ac adsorbed on the membranes was calculated from the difference of radioactivity in solution before (*A*_0_) and after equilibration (*A*_eq_). The distribution coefficient, *K*_d_, was calculated using eq 4

4 $$K_{d}=\frac{A_{0}-A_{\mathrm{eq}}}{A_{0}}\left(\frac{V}{m}\right)$$

Where *A*_0_ is the initial radioactivity of ^225^Ac (μCi), *A*_eq_ is the radioactivity of ^225^Ac at equilibrium, *V* is the volume of the solution (mL), and *m* is the mass of the membrane (mg).

### 3.15 THDGA Membrane Dynamic Desorption: ^225^Ac

For the dynamic desorption experiments, ^225^Ac was adsorbed onto THDGA membranes similarly to the La­(III) adsorption experiments. For each experiment, 1 mL of the ^225^Ac-containing solution was pipetted into a sample vial (Wheaton sample 6 mL HDPE, polypropylene caps) containing 1.25 mg THDGA membrane. The sample was immediately analyzed via gamma spectroscopy to quantify the initial radioactivity of ^225^Ac. Then, the THDGA membrane was equilibrated with the ^225^Ac solutions for 3 h on a shaker table at 100 rpm. After, the membrane was separated from the supernatant via centrifugation at 12000 rpm for 10 min.

Dynamic desorption experiments were performed by placing the ^225^Ac loaded THDGA membrane in a syringe filter and eluting ^225^Ac from the fibers with 10 M nitric acid. Prior to the experiment, the 45 μm PTFE syringe filter (Phenomenex Phenex, 25 mm diameter) was prewetted first with ethanol and then with 4 M nitric acid. It was then superglued (LocTite 414) to a stopcock with 1.6 mm inner diameter C-Flex Ultra pump tubing (Masterflex). To set up the experiment, the ^225^Ac-adsorbed THDGA membrane sample was placed in the syringe filter, and a 60 mL syringe (sans plunger) was attached to the membrane-containing syringe filter. The supernatant was pipetted into the syringe. A peristaltic pump (Cole-Parmer Masterflex 07525–20, 12 mL/h) was used to remove the ^225^Ac loading fraction from the filter housing. Then a series of 1 mL fractions of 10 M nitric acid were placed into the syringe and pumped through the membrane. In between fractions, the membrane was purged with air using the peristaltic pump. The loading and desorption fractions were collected into separate sample vials. Control samples without THDGA membranes were performed similarly.

^225^Ac quantification was performed as described in Section 3.14. ^225^Ac desorption from the THDGA membranes was calculated fraction-by-fraction by the difference between the THDGA membrane samples and the control samples.

### 3.16 Statistical Analysis

A statistical analysis was performed on the model fitting parameters, *Q*_max_ and *K*_L_, resulting from fitting the data with the Langmuir isotherm. For this statistical analysis, three sets of replicate data were modeled with the Langmuir isotherm for each acid condition. Then, the resulting model parameters were compared using an analysis of variance (ANOVA) with Tukey’s posthoc test while assuming equal variances. Two sets of ANOVA testing were performed: 1) for each support type, comparing the means of *Q*_max_ and *K*_L_ grouped by acid strength (4 vs 6 vs 10 M nitric acid), and 2) for each acid strength comparing the *Q*_max_ and *K*_L_ grouped by support type (resin vs membrane). All analyses were conducted with a significance level of *p* < 0.05. Nonlinear curve fitting was performed in OriginLab Software (version 2023b). Statistical analyses were performed in Minitab (version 22.3).

## 4 Results and Discussion

### 4.1 Synthesis and Characterization of aTHDGA Ligand

The amine-terminated diglycolamide (aTHDGA) was synthesized in four steps according to Figure 1. In the first step, 1,6-hexanediamine was monoalkylated by reaction with bromohexane, and in the second step the primary amine was selectively protected using the trifluoroacetate group. Then a mixture of the monoprotected diamine and dihexylamine was reacted with diglycoloyl chloride and, after a crude workup, the terminal amine was deprotected. The desired monoamine product was isolated via column chromatography in 14.5% cumulative yield as the trifluoroacetate ammonium salt. High-resolution mass spectrometry and ^1^H, ^13^C, and ^19^F NMR spectroscopy are indicative of the pure aTHDGA product; NMR spectra are shown in the Figures S1–S3. ^1^H and ^13^C NMR spectra show multiplicities consistent with amide rotational isomerism which did not coalesce at temperatures up to 100 °C (data not shown).

### 4.2 Formation and Characterization of THDGA Membranes

aTHDGA ligands were covalently tethered to electrospun PVBC membranes in a single step reaction, Figure 2A. The morphology of the PVBC membranes (before the reaction) and the THDGA membranes (after the reaction) were characterized, Figure 2B,C.

**Figure 2:**
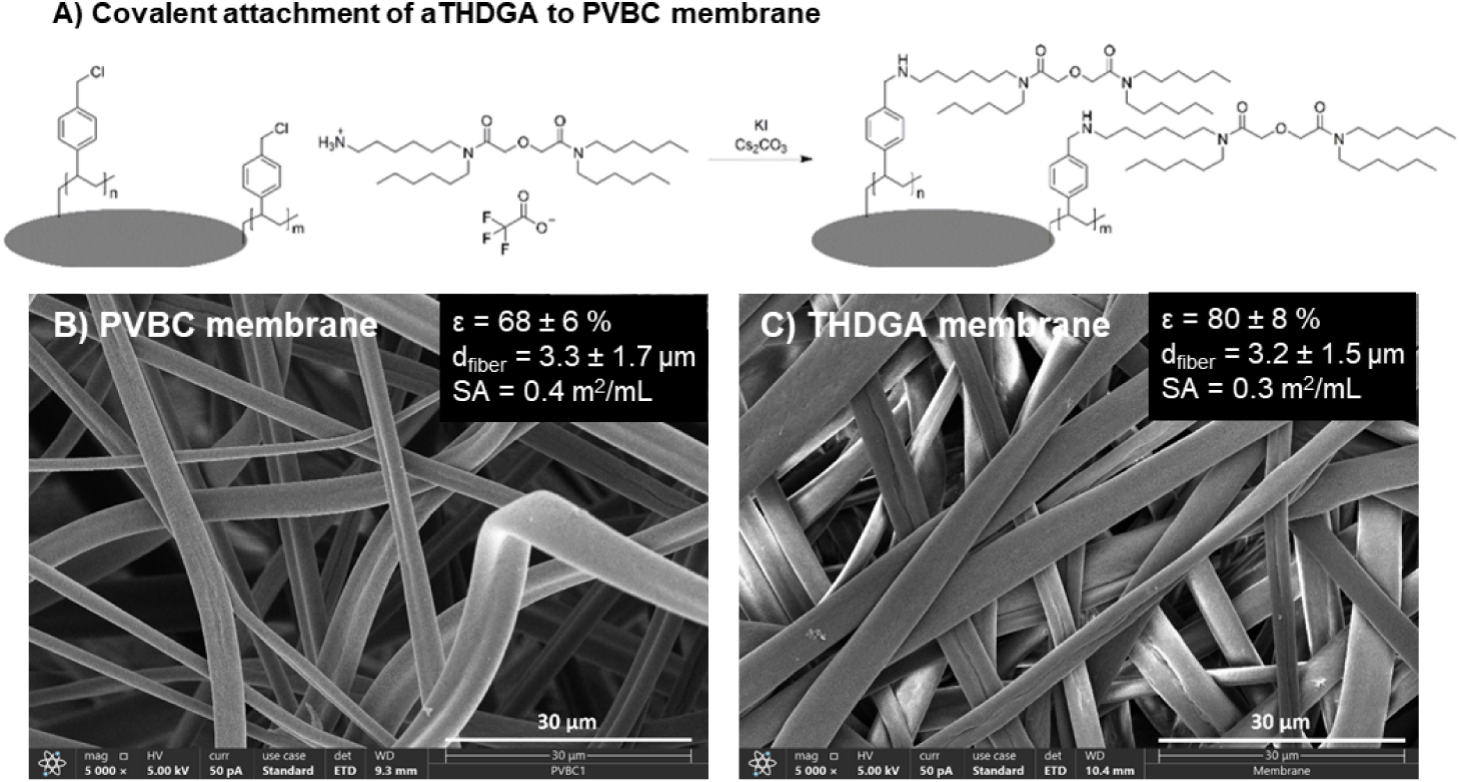
(A) Reaction scheme for the covalent attachment of aTHDGA to an electrospun PVBC membrane. SEM images and morphological characteristics of porosity (ε), fiber diameter (*d*_fiber_), and surface area (SA) of fibers for (B) PVBC membranes and (C) THDGA membranes. The surface area was calculated according to Lavoie et al.^35^ using the measured porosity and fiber diameter from this work.

SEM images of the electrospun unreacted PVBC membranes show randomly oriented fibers with a diameter of 3.3 ± 1.7 μm, Figure S5. After the reaction with aTHDGA, the membranes remain intact and exhibit discrete fibers with a diameter of 3.2 ± 1.5 μm, Figure S6. In contrast, the diameter of a TODGA resin is 67 ± 13 μm, Figure S7. The porosity of the PVBC and THDGA membranes were measured at 68 ± 6% and 80 ± 8%, respectively. According to the analysis by Lavoie et al.,^35^ which describes the relationship between fiber diameter, porosity, and surface area in nonwoven membranes, the THDGA membrane (3.2 μm diameter, ∼80% porosity) is estimated to have a surface area of 0.25 m^2^/mL. Similarly, the PVBC membrane (3.3 μm diameter, ∼68% porosity) is estimated to have a surface area of approximately 0.39 m^2^/mL. These results suggest that the PVBC membranes have a surface area approximately 1.6× greater than the THDGA membranes per unit volume. Although the THDGA membranes maintain a similar fiber diameter and a comparable surface area to the unreacted PVBC membranes, the higher porosity (∼80%) results in a lower fiber density and therefore lower surface area on a per volume basis. The pure water permeance (*A*) for each membrane was calculated from the slope of the flux (*J*_w_) versus transmembrane pressure (Δ*P*), according to eq 5.

5 $$J_{w}=A\Delta P$$

The permeance of the PVBC membranes was 111,800 ± 19,100 LMH/bar, while the THDGA membranes exhibited a permeance value of 121,000 ± 12,800 LMH/bar, Figure S8. Both membranes have a high permeance which is consistent with other electrospun membranes. When compared using a student’s *t* test at the 95% confidence interval, the permeances are not statistically different (*p* > 0.05). Thus, the minor differences in the PVBC and THDGA membrane morphology do not affect the membrane permeance. While a high permeance is necessary for short filtration times at low applied pressures, the maximum operational flow rate will be dictated by the complexation kinetics of the metal with the THDGA membranes.

Chemical analysis of the THDGA membranes was performed using ATR-FTIR, Figure 3. ATR-FTIR of the THDGA membrane shows the absence of the CH_2_Cl wag at 1265 cm^–1^, the absence of the trifluoroacetate C=O stretch (1678 cm^–1^), and the appearance of the amide C=O stretch at 1642 cm^–1^. XPS is consistent with ATR-FTIR, showing absence of chlorine, fluorine, as well as the absence of cesium and iodine on the surface of the membranes, Figure S4. These results are consistent with the surface of the membrane fibers being coated with covalently bound aTHDGA via the S_N_2 mechanism and the counterions from the reaction mixture having been successfully washed away.

**Figure 3:**
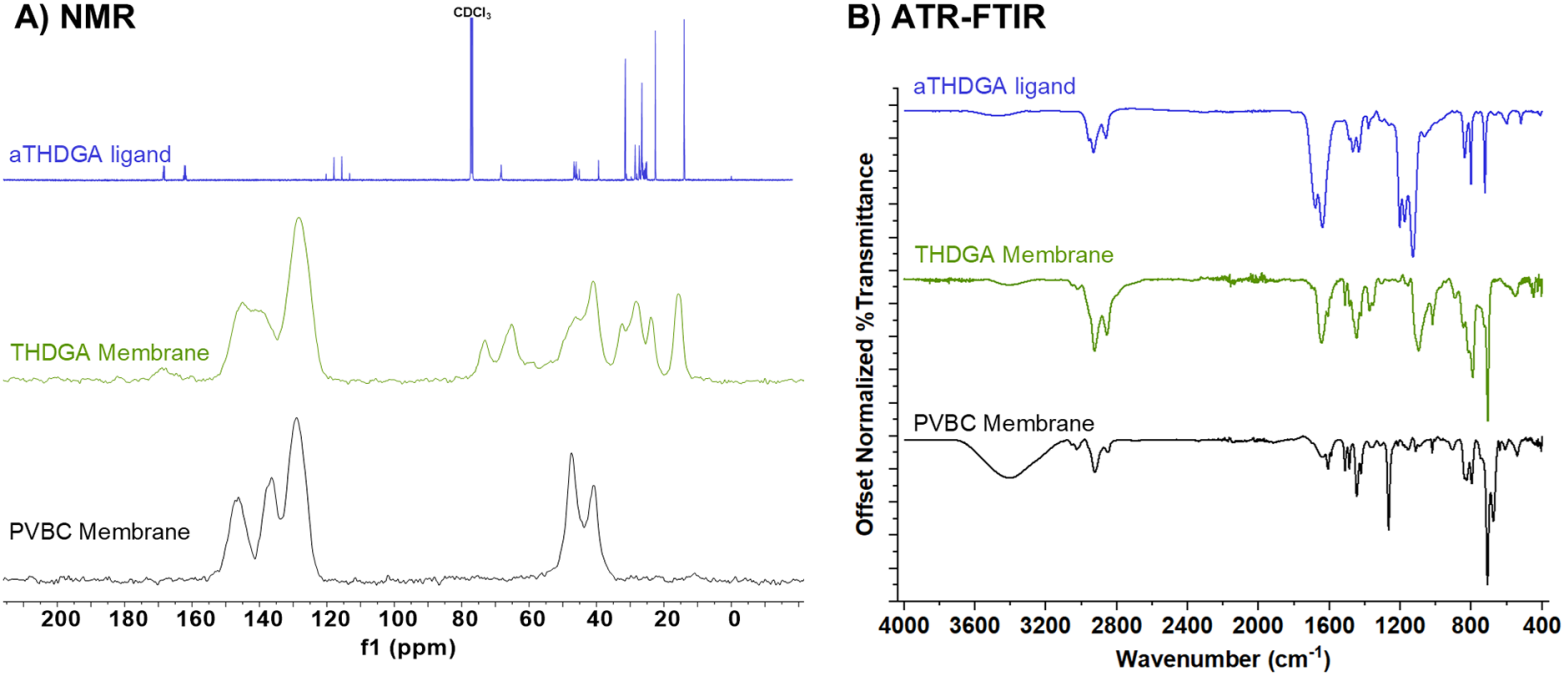
Characterization of the PVBC membrane, aTHDGA ligand, and THDGA membrane by (A) ^13^C NMR and (B) ATR-FTIR.

Further analysis of the THDGA membranes was performed by solid state NMR (Figure 3), which shows a commensurate proportion of aTHDGA in comparison to PVBC. The presence of the amide carbonyl at 168 ppm is discernible in the THDGA membrane spectrum. In the aTHDGA spectrum, the set of peaks near 68 ppm (amide rotational isomerism gives rise to multiple peaks) is attributable to the ethereal CH_2_OCH_2_ carbons. In the THDGA membrane spectrum, the CH_2_OCH_2_ peak from the ligand is observed in the region 60–80 ppm. The appearance of a second peak in this region is consistent with covalent attachment of aTHDGA to the benzylic sites of the membrane. The relative downfield position of this peak would even be consistent with cross-linking, one aTHDGA reacting at two or three benzylic sites. The insolubility of the DGA membranes in organic solvents, compared to the ready solubility of the unreacted PVBC membranes, is also consistent with such cross-linking.

### 4.3 Equilibrium Adsorption – La­(III) And ^225^Ac

Batch equilibrium adsorption experiments for TODGA resins and THDGA membranes were modeled with the Langmuir isotherm for 4, 6, and 10 M nitric acid matrices, shown in Figures S9 and S10, respectively. Both adsorbents exhibited a characteristic saturation curve. As an example, the data and model fits for La­(III) adsorption on TODGA resins and THDGA membranes from 10 M nitric acid are shown in Figure 4A,B, respectively. The Langmuir isotherm model fitting parameters, *Q*_max_ and *K*_L_, for TODGA resins and THDGA membranes at all tested acid concentrations are summarized in Figure 5 and Table S3.

**Figure 4:**
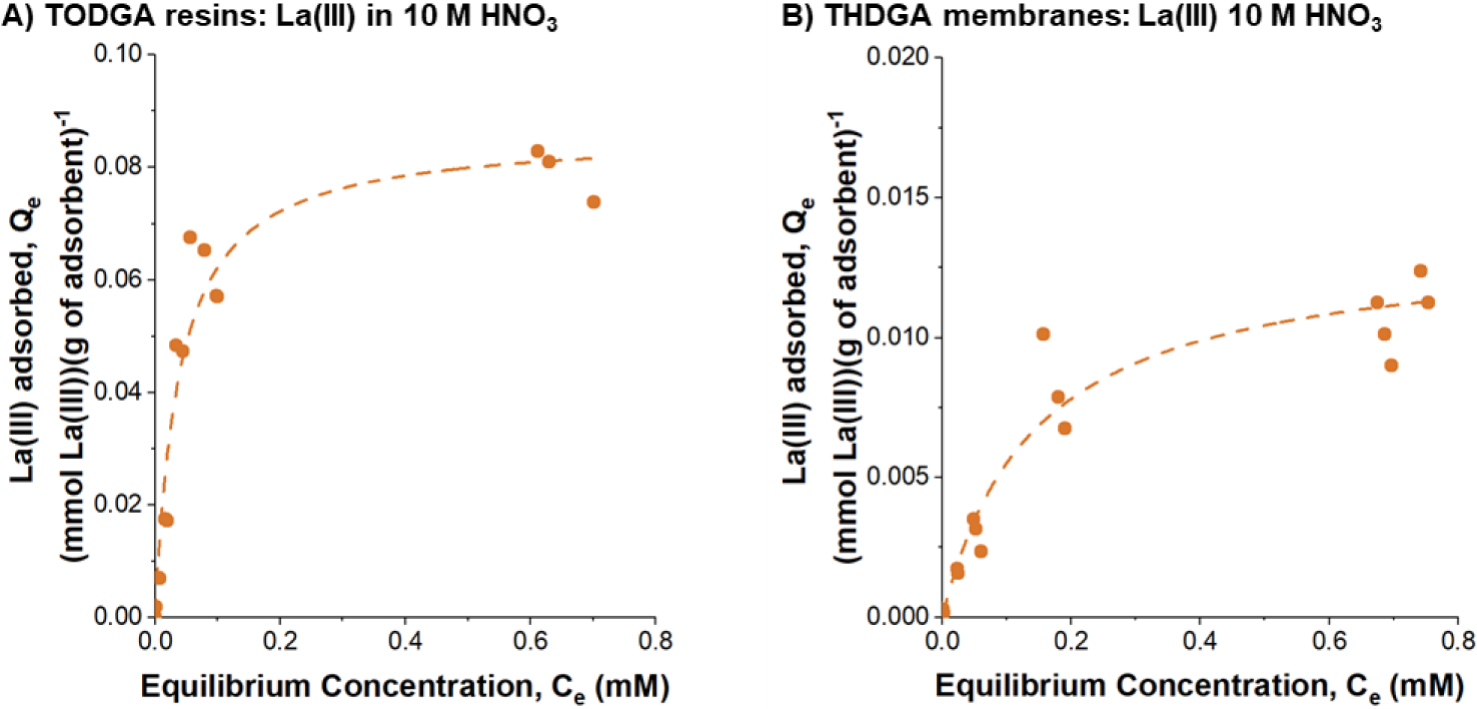
Equilibrium adsorption curves for (A) La­(III) in TODGA resins and (B) THDGA membranes in 10 M nitric acid. Model line (- -) represents the Langmuir isotherm model.

**Figure 5:**
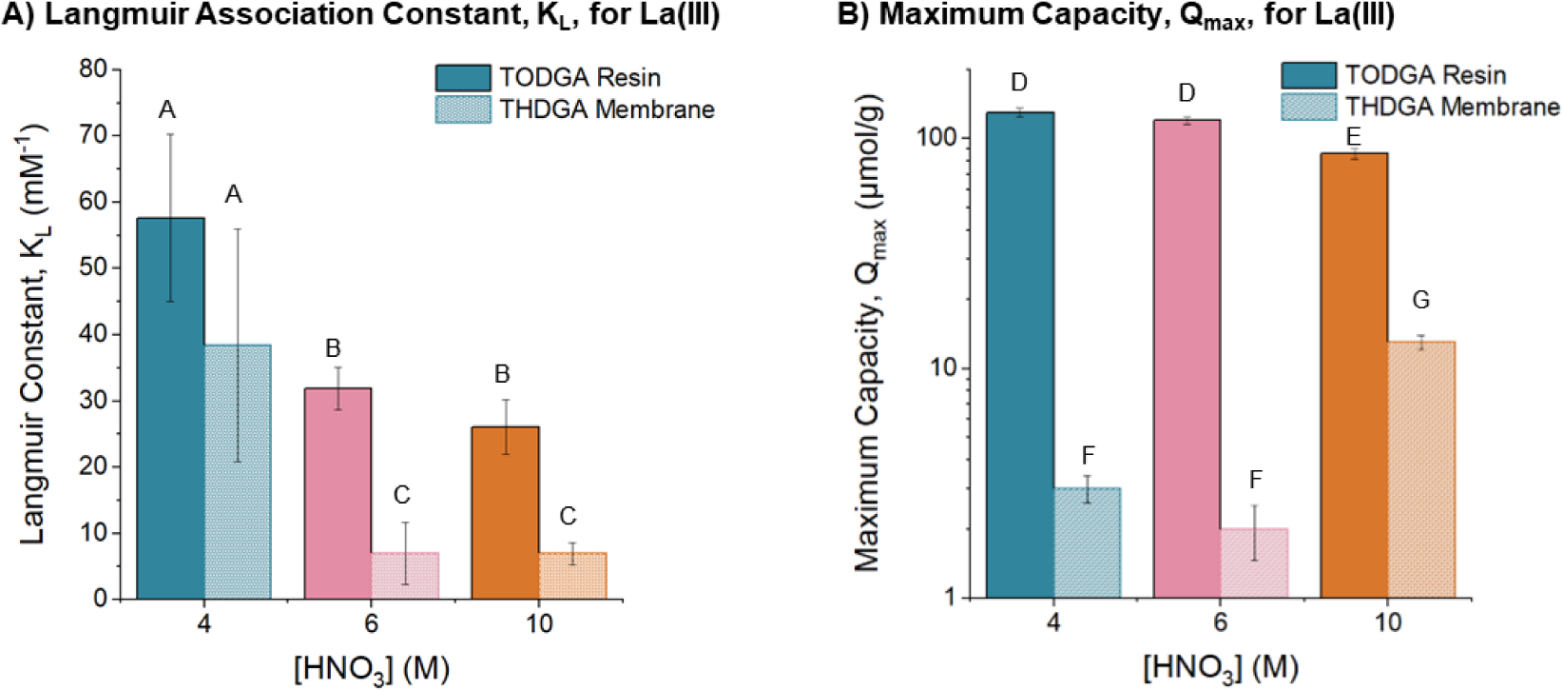
Langmuir model parameters for La­(III) adsorption on TODGA resins and THDGA membranes: (A) Langmuir association constant, *K*_L_ and (B) Maximum capacity, *Q*_max_. Solid bars correspond to TODGA resins and dotted bars correspond to THDGA membranes for 4 M HNO_3_ (teal), 6 M HNO_3_ (pink), and 10 M HNO_3_ (orange). Bars designated with matching letters were determined to not be statistically different (*p*-value >0.05). Bars designated with different letters were determined to be statistically different (*p*-value <0.05).

First, the THDGA membranes exhibit consistent affinity trends with TODGA resins as measured by the Langmuir association constant, *K*_L_, for La­(III) adsorption. Both materials show a decrease in affinity with increasing nitric acid concentration. In 4 M nitric acid, the affinity of the resins and membranes for La­(III) is comparable and statistically not different (*p* > 0.05). In both 6 and 10 M nitric acid, the affinity of the THDGA membrane is significantly lower than the resins (*p* < 0.05).

The trends in maximum capacity (*Q*_max_) differ significantly between the resins and membranes. The resins show a statistically significant and lower capacity in 10 M nitric acid than in 4 and 6 M nitric acid (*p* < 0.05). The membranes, however, show the opposite trend where the capacity is higher in 10 M nitric acid than in 4 and 6 M nitric acid. Overall, the resins exhibit significantly higher capacities than the membranes across all tested acid concentrations. Importantly, control studies were performed using the PVBC membranes and all exhibited negligible adsorption of La­(III), Figure S13.

Due to the limited supply of ^225^Ac for this work, it was not possible to construct full saturation curves and model them with the Langmuir isotherm to determine the *Q*_max_ and *K*_L_. Instead, adsorbent dosing experiments were performed with a fixed initial activity of ^225^Ac (8–12 μCi) and variable membrane mass, Figure 6A. In both 4 and 6 M nitric acid, the amount of adsorbed ^225^Ac increased linearly with increasing membrane mass. More ^225^Ac adsorbed to the membranes at 4 M nitric acid than at 6 M nitric acid. At 10 M nitric acid, the ^225^Ac adsorbed to the THDGA membranes was negligible.

**Figure 6:**
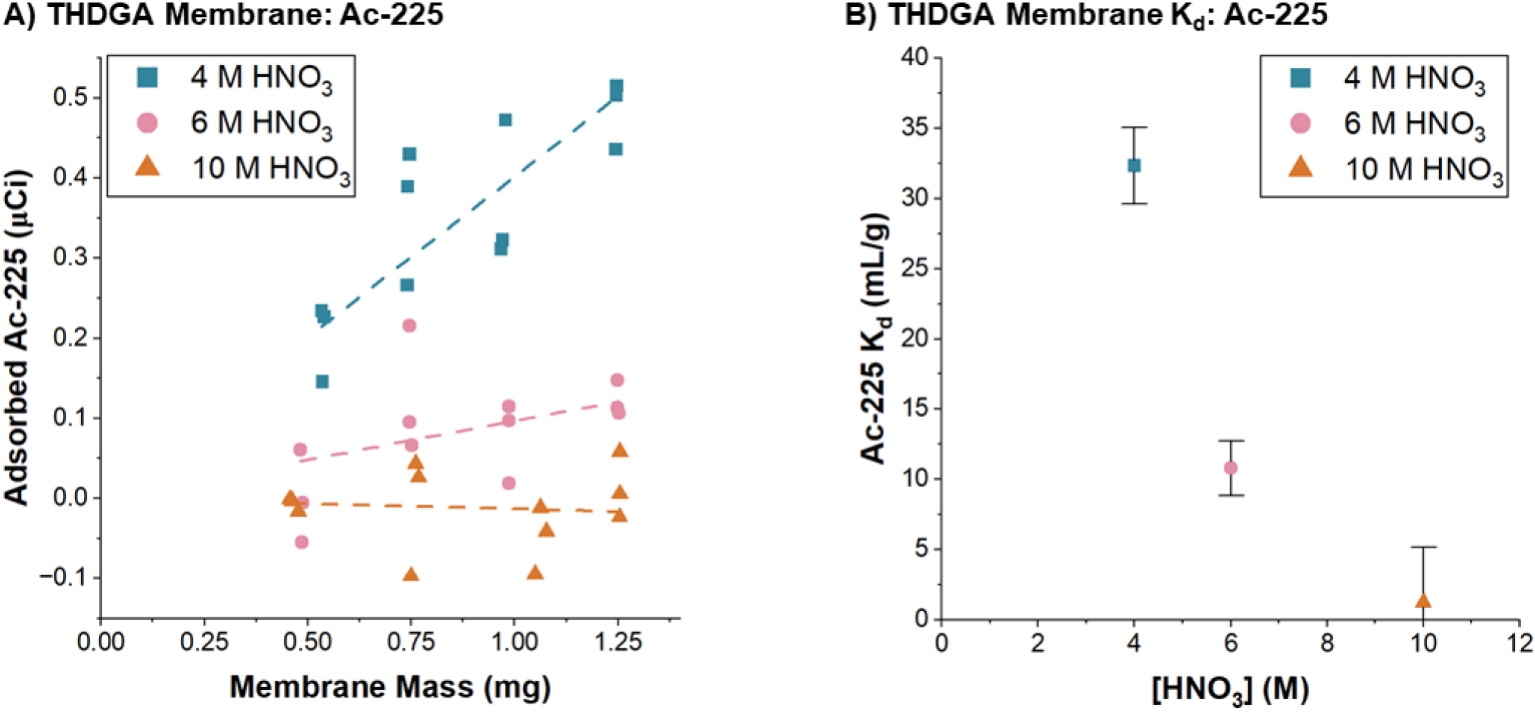
(A) Equilibrium adsorption of ^225^Ac on THDGA membranes with initial radioactivity of 12 μCi of ^225^Ac and increasing mass of THDGA membrane, and (B) Distribution coefficients of THDGA membranes calculated for an initial radioactivity of 12 μCi of ^225^Ac and THDGA membrane mass of 1.25 mg.

The data points for the trials using 1.25 mg of THDGA membrane were converted to distribution coefficients, eq 4, and replotted in Figure 6B. These trends are consistent with the adsorption of ^225^Ac on TODGA resin reported previously by Radchenko et al.^36^ Thus, despite significantly lower adsorption capacities as measured for La­(III), THDGA membranes follow the trends in affinity expected for ^225^Ac.

Batch equilibrium adsorption experiments with La­(III) revealed that the THDGA membranes have 10% of the maximum capacity of the TODGA resins. While the trend in affinity for La­(III), shown by *K*_L_, were consistent between the TODGA resins and the THDGA membranes—the magnitude of the affinity for THDGA membranes is significantly lower than the resins in stronger nitric acid concentrations (6 and 10 M). This difference in affinity appears to be a unique feature of immobilizing the THDGA on a polymer surface. When dissolved in *n*-octane for solvent extraction, THDGA and TODGA ligands exhibit negligible differences in partition coefficients.^37^ For the THDGA membranes, differences in adsorption at higher nitric acid concentrations may imply a difference in coordination modes between the surface-bound DGA of the membranes and the physisorbed DGA of the resins. However, without knowing the surface bound DGA-La­(III) species, it is not possible to provide direct evidence of the root cause for the differences between the resin and the membrane.

### 4.4 Modeled Selectivity Analysis: La­(III)/^225^Ac

In the absence of adsorption experiments performed with binary mixtures, the selectivity of an adsorbent material can be calculated from the ratio of distribution coefficients. The distribution coefficients are typically measured when the ligand is in excess of the adsorbing metal. For example, the original distribution coefficients for the TODGA resins were collected using a molar ratio of ligand to adsorbate of 50:1.^15^ However, at the time of the ^225^Ac adsorption experiments, the THDGA concentration in the membranes was not known. The discussion herein will focus on the “as synthesized” materials for TODGA resins. The ligand loading of the TODGA resins is known to be 40 wt %.^15^

Without La­(III) radiotracers, experimentally determining the La­(III)/^225^Ac selectivity of the THDGA membranes is not possible due to the low molar concentrations of La­(III) required (pmol/mL). A key benefit to modeling the saturation curves with isotherm models is the ability to predict distribution coefficients at different conditions (i.e., adsorbent mass, initial adsorbate concentration). The estimated distribution coefficient for La­(III) on the THDGA membranes was modeled to match the ^225^Ac adsorption experiments performed at BNL experiments (*C*_0_ = 12 μCi/mL or 0.88 pmol/mL, *V* = 1 mL, *m* = 1.25 mg). The distribution coefficient for La­(III) was calculated in two ways: 1) using the Langmuir isotherm to calculate the *K*_d_ for the given adsorbent mass, volume, and initial activity and 2) using the linear adsorption isotherm to calculate the *K*_d_ for the same adsorbent mass, volume, and initial activity. Then, the La­(III)/^225^Ac selectivity was calculated by dividing the *K*_d_ of each species.^28^ The results of the *K*_d_ and selectivity calculations using both isotherm models are shown below, in Table 1. Using either isotherm, the THDGA membranes generally follow the same trend of increasing selectivity for ^225^Ac with increasing acid concentration. These trends are consistent with the TODGA resins in which both species are loaded and washed at lower acid concentration (4 and 6 M nitric acid). During elution with 10 M nitric acid, the resins and membranes must release ^225^Ac while retaining La­(III) and therefore high selectivity for La­(III) is desired.

**Table 1:** Modeled Selectivity for THDGA Membranes All Experiments were Performed at the Same Conditions as ^225^Ac Adsorption Experiments (*C*_0_ = 0.88 pmol/mL, *V* = 1 mL, *m* = 1.25 mg)^a^

	Calculated via Langmuir Isotherm		Calculated via Linear Isotherm	
Acid	*K*_d,La_ (mL/g)	Selectivity (*K*_d,La_/*K*_d,Ac_)	*K*_d,La_ (mL/g)	Selectivity (*K*_d,La_/*K*_d,Ac_)
4 M HNO_3_	115	3.6	39.4	1.2
6 M HNO_3_	14	1.3	33.7	3.1
10 M HNO_3_	90	73.0	68.1	56.8

a The calculations represent equimolar mixtures of La­(III) and ^225^Ac.

The selectivity of the THDGA membranes can be benchmarked against the TODGA resins at 4 and 6 M HNO_3_ by taking the quotient of literature reported capacity factor (*k*’) values for La and Ac.^15,36,38^ The capacity factor is proportional to the *K*_d_ and accounts for resin-specific information such as the resin density and ligand loading.^28^ Using the capacity factors from Horwitz et al.,^15^ the selectivity for the TODGA resins in 4 M HNO_3_ and 6 M HNO_3_ is 7 and 24, respectively. The distribution coefficient for ^225^Ac was measured by Radchenko et al.^36^ in 10 M HNO_3_; however, they do not report the analogous data for La. One study reports the *k*’ for La adsorption to TODGA resins in 10 M HNO_3_, but the resolution of the plots were too low to reliably digitize.^39^ To make a comparison with the literature data, we used the linear portion of the saturation isotherm measured in this work (Table S4) to calculate the *K*_d_ of La at the same conditions as the Radchenko ^225^Ac adsorption experiments. That value is 1,010 mL/g. Dividing the calculated *K*_d,La_ by the experimental *K*_d,Ac_ (67 mL/g)^36^ yields a selectivity of 15 for La over ^225^Ac in 10 M nitric acid. Regardless of the isotherm used for modeling, the THDGA membrane selectivity is >55 in 10 M HNO_3_ for La over ^225^Ac which is higher than the TODGA resins. These calculations suggest that THDGA membranes may have sufficient selectivity for the desired separation. Future experimental work will be conducted to directly measure the selectivity.

### 4.5 Desorption: ^225^Ac and La­(III)

THDGA membranes with adsorbed ^225^Ac were placed in a syringe filter and eluted with 10 M nitric acid at a flow rate of 0.2 mL/min to match that of the resin column chromatography method for ^225^Ac purification.^36^ The desorption curve which depicts the adsorbed activity as a function of elution time is shown in Figure 7. Complete elution of the ^225^Ac is achieved after 20 min (4 mL) of 10 M nitric eluent is passed through the column. To compare with the state-of-the-art, ^225^Ac elution data collected by Radchenko et al. were reanalyzed. Unfortunately, there was no such elution data for the TODGA resin (linear DGA resin), so the comparison is made with the TEHDGA resin (branched DGA resin). In the original work, 2.94 mCi of ^225^Ac was loaded onto the column and eluted using 10 M nitric acid. The elution profile was presented as the radioactivity of each eluent fraction versus bed volume. To compare with the current work, the data was transformed into a desorption curve using the volumetric flow rate (0.2 mL/min) and bed volume (0.3 mL), Figure 7B. The resulting data fitted with an exponential decay model for desorption, eq 6

6 $$A\left(t\right)=A_{0}\exp\left(-k_{\mathrm{off}}\right)$$

Where *A*_0_ is the activity loaded on the resin or membrane, *A­(t)* is the activity at any time, *i*, and *k*_off_ is the rate constant of desorption.

To benchmark the THDGA membrane elution time and volume against the TEHDGA resins, it was necessary to choose an arbitrary and nonzero end point in the elution curve. Comparing the time to elute 98% of *A*_0_ from the resin and membrane (2.94 mCi) removes some uncertainty associated with identifying a “zero” term for an exponential decay model. Under these criteria, the elution time for the TEHDGA resin was 160 min (pink dots). Applying the calculated desorption rate constant (*k*_off_ = 0.140 ± 0.002 min^–1^) and the exponential model with *A*_0_ = 3 mCi predicted an elution time of 28 min from the THDGA membrane. In addition to the time savings, the fast desorption from the membrane adsorber surface yields a more concentrated product (6 mL). By these preliminary estimates the DGA membranes have the potential to reduce elution time and volume by a factor of 5; however, there are several caveats to this work. The scaling up calculation does not account for the inevitable increased bed volume required to accommodate a higher mass of membrane which may impact the elution volume. The present data uses the THDGA membrane (linear, 6 carbon DGA) whereas Radchenko et al. used the DGA branched resin (branched, 8 carbon DGA). Future experiments will focus on clarifying these factors.

Similarly, elution experiments were performed using the La­(III)-loaded THDGA membranes. Elution was attempted using pH 2 nitric acid at a flow rate of 0.5 mL/min. This pH was chosen because the commercial TODGA resins have a low distribution coefficient for La­(III) in near-neutral pH nitric acid solutions.^15^ This is consistent with additional literature reports that identify nitrate and hydronium ions in the binding mechanism of lanthanides to DGA ligands in solvent extraction systems.^40–42^ In all 30 fractions collected over the 1 h elution period, the La­(III) concentration in the eluent was below the ICP-OES detection limit as shown in Figure S14. To further investigate the irreversible adsorption of La­(III), time-of-flight secondary ion mass spectrometry (ToF-SIMS) was performed on the surface of the THDGA membranes, shown in Figure S15. Spectra were collected before and after elution and confirmed that La­(III) remained bound to the surface of the THDGA membranes even after elution.

**Figure 7:**
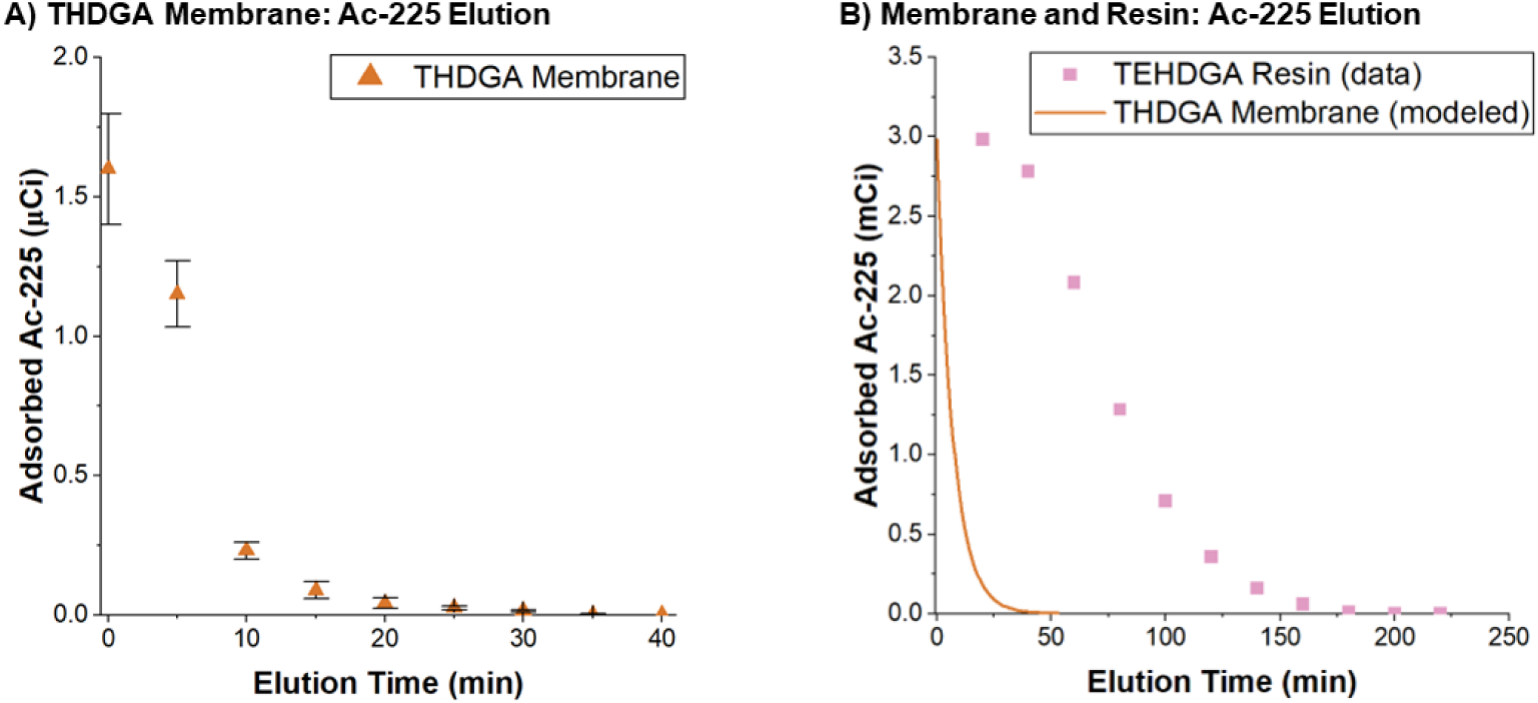
(A) ^225^Ac desorption from THDGA membranes. The initial activity on the fibers was 1.6 μCi as calculated by a mass balance. Full elution of the fibers is completed after 20 min (4 mL) of 10 M nitric acid is passed through the membrane filter at 0.2 mL/min. Error bars represent the standard deviation of triplicate measurements made with membrane fibers loaded in syringe filters. (B) ^225^Ac desorption data^36^ from TEHDGA resin (pink squares) and modeled desorption from THDGA membranes (orange line).

Gujar et al.^43^ and Ansari et al.^44^ prepared extraction chromatography resins in which multipodal DGA ligands were impregnated onto Chromosorb-W supports. These DGA-based resins were loaded with Eu­(III) from 3 M HNO_3_ and subsequently stripped using low-molar ethylenediaminetetraacetic acid (EDTA) solutions, with 0.01 M EDTA at pH 2–4 requiring approximately 4–10 mL of eluent depending on the resin formulation. Similarly, Shusterman et al.^23^ developed a covalently grafted DGA onto mesoporous silica (SBA-15) and showed that a 1 mM EDTA eluent fully desorbed Am­(III) and Eu­(III). These previous studies confirmed that eluents like EDTA can effectively desorb trivalent lanthanides and actinides from DGA-functionalized adsorbents. Since the THDGA membranes also employ covalently bound DGA ligand, EDTA-assisted elution may be feasible for these materials and future work will explore this approach. Practically, the apparent irreversible adsorption of La­(III) will not impact ^225^Ac purification; however, it does have implications for the potential regeneration and reuse of the membranes. Further, these results suggest that La­(III) exhibits a binding mechanism on THDGA membranes that is distinctly different from the TODGA and TEHDGA resins. Future studies will be conducted using competitor ions (Ce, Ba, Ra) that are present during the final ^225^Ac purification using the THDGA memrane column.

## 5 Conclusions

In this work, an aTHDGA ligand was synthesized, characterized, and covalently tethered to an electrospun PVBC fiber mat to create a THDGA membrane adsorber. Batch equilibrium adsorption experiments were performed using La­(III) and ^225^Ac in 4, 6, and 10 M nitric acid which were selected to mimic the loading and eluting conditions of a resin-packed column used to purify accelerator-produced ^225^Ac. Surface characterization by XPS, ATR-FTIR, and solid-state NMR support the covalent attachment of the THDGA to the PVBC membranes.

The development of the THDGA membrane adsorbers was motivated by the need for rapid purification and low elution volumes when purifying accelerator-produced ^225^Ac. A major conclusion of this work is that while the THDGA membrane adsorbers exhibit similar trends to the TODGA resins; they generally exhibit weaker binding and lower capacities. Despite these differences, the THDGA membranes exhibit a La­(III)/^225^Ac selectivity of 57 in 10 M nitric acid and rapid desorption of ^225^Ac at relevant elution conditions for ^225^Ac purification—achieving desorption in less than 20 min. Preliminary modeling suggests that the THDGA membranes have the potential to decrease elution times and volumes when compared to the TEHDGA resins. Future work will continue the full characterization of the THDGA membranes with additional competitor ions and pursue the purification of ^225^Ac from irradiated targets. This platform technology has the potential to enable rapid purification of trivalent lanthanides and actinides for a variety of applications.

## Supplementary Material



## Notes

Synthesis protocols, SEM images, saturation curves for THDGA membranes and TODGA resins, model fitting parameters, statistical analyses, and example calculations for the selectivity analysis (PDF)
