# Supplementary material for: Toward Rapid Actinium-225 Purification via Membrane Adsorbers with Covalently Tethered Diglycolamide Ligands
Source: ACS Appl Mater Interfaces. 2026 Feb 5;18(6):10464–75. doi: 10.1021/acsami.5c17147 (PMC12926944; doi:10.1021/acsami.5c17147)
Supplement: Supplementary file 1 [file am5c17147_si_001.pdf]

Supporting Information

Towards Rapid Actinium-225 Purification via Membrane Adsorbers with Covalently Tethered Diglycolamide Ligands

Shruti Krishna Radhakrishnan,[a§] Megan M. Sibley,[a,§] Bernadette L. Schneider,[a] Pavithra H. A. Kankanamalage,[b] Tuli Banik,[a] Tae Kyong John Kim,[d] Jasmine Hatcher-Lamarre,[b] Luke A.F. Venturina[a], Timothy Yen,[a] Joshua T. Damron,[c] Alec Johnson,[a] Alexa G. Ford,[a] Trent Kozar,[a] Tugce Uz,[d] Weimin Zhou,[b] Cathy S. Cutler,[b] Christine E. Duval[a*]

[a] Department of Chemical & Biomolecular Engineering, Case Western Reserve University, Cleveland, OH 44106, United States

[b] Isotope Research and Production Department, Brookhaven National Laboratory, Upton, NY 11973, United States

[c] Chemical Sciences Division, Oak Ridge National Laboratory, Oak Ridge, TN 37831, United States

[d] Swagelok Center for Surface Analysis of Materials, Case Western Reserve University, Cleveland, OH 44106, United States

*Email: christine.duval@case.edu

§ Co-first authors

Contents

Synthesis of aTHGA ligand and chemical characterization ………. page 3

General Synthesis Information ………. page 3

Figure S1: ^{1}H NMR of aTHDGA ………. page 5

Figure S2: 13C NMR of aTHDGA ………. page 6

Figure S3: 19F NMR of aTHDGA ………. page 7

Figure S4: XPS of PVBC, aTHDGA ligand, THDGA membrane ………. page 8

Resin and Membrane Morphology

Figure S5: SEM images of PVBC membranes ………. page 9

Figure S6: SEM images of THDGA membranes ………. page 9

Figure S7: SEM images of TODGA resins ………..page 10

Table S1: Porosity and fiber diameter for THDGA membranes ………. page 11

Table S2: Porosity and fiber diameter for PVBC membranes ……….page 11

Membrane Permeance

Figure S8: Pure water flux versus transmembrane pressure ……….page 12

Adsorption and Desorption Experiments

Figure S9: Equilibrium adsorption plot for TODGA resins ……….page 13

Figure S10: Equilibrium adsorption plot for THDGA membranes ……….page 14

Table S3: Model fitting parameters for Langmuir isotherm ……….page 15

Figure S11: Linear isotherms for TODGA resins ……….page 16

Figure S12: Linear isotherms for THDGA membranes ……….page 17

Figure S13: Equilibrium adsorption data for PVBC membranes ……….page 19

Figure S14: La(III) desorption on THDGA membrane ……….page 20

Figure S15: ToF-SIMS spectra of THDGA elution experiments ……….page 20

Statistical Analysis of Adsorption Experiments ………page 21

Calculations Supporting the Selectivity Estimations ………page 41

Synthesis of aTHDGA Ligand

General Synthesis Information

All reagents and solvents were of reagent quality and were used as received. ^{1}H, ^{13}C, and ^{19}F NMR spectra were recorded using a Bruker Ascend 500 MHz spectrometer with Prodigy liquid nitrogen cryoprobe. Chemical shifts δ (in ppm) for ^{1}H spectra are referenced to tetramethylsilane using the residual protio-solvent as an internal standard. Coupling constants (*J*) are expressed in hertz (Hz). All syntheses and purifications were performed at ambient conditions unless otherwise specified.

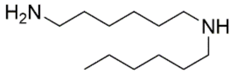

N-hexyl-1,6-hexanediamine

Hexanediamine (17.545 g, 150.98 mmol) was dissolved in THF (200 mL) and hexyl bromide (5.004 g, 30.31 mmol) was added. The reaction mixture was stirred at reflux for two days. Then, after cooling to room temperature, dichloromethane (100 mL) was added, and the mixture was washed three times with water. The organic layer was dried via rotary evaporation, yielding a white solid (6.262 g) and was used without further purification. ^{1}H NMR (500 MHz, DMSO-d6): δ = 2.88 (br s, 1H), 2.44 (t, *J*=7.0 Hz, 4H), 1.39 – 1.31 (m, 7H), 1.29 – 1.22 (m, 11H), 0.86 (t, *J*=7.0 Hz, 3H).

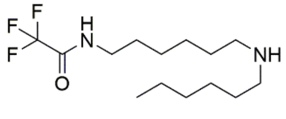

N-(6-trifluoroacetamidohexyl)-*N*-hexylamine

N-hexyl-1,6-hexanediamine (5.113 g, 25.52 mmol) was dissolved in dichloromethane (150 mL) and ethyl trifluoroacetate (3.990 g, 28.08 mmol) was added. The reaction mixture was stirred at room temperature for 24 hours and then dried via rotary evaporator, yielding a brown oil (8.319 g) which was used without further purification. ^{1}H NMR (500 MHz, $CDCl_3$): δ = 6.66 (br s, 1H), 3.36 (q, *J*=6.5 Hz, 2H), 2.72 (q, *J*=7.3 Hz, 4H), 1.59 (quint, *J*=6.9, 6H), 1.41 – 1.35 (m, 4H), 1.34 – 1.26 (m, 7H), 0.88 (t, *J*=6.8 Hz, 3H).

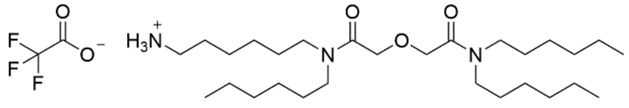

N-(6-aminohexyl)-*N*,*N*',*N*'-trihexyl diglycolamide trifluoroacetate

Diglycolic acid (3.201 g, 23.87 mmol) was dissolved in 50 mL dry tetrahydrofuran (stored over 3 Å molecular sieves) and thionyl chloride (1 M in dichloromethane, 53 mL, 53 mmol) was added at room temperature. The solution was stirred at reflux for one hour. Then a solution containing dihexylamine (4.886 g, 26.36 mmol), *N*-(6-trifluoroacetamidohexyl)-*N*-hexylamine (7.813 g, 26.36 mmol), and triethylamine (7.283 g, 71.97 mmol) in dry tetrahydrofuran (100 mL) was added. The reaction mixture was stirred at reflux for 24 hours. The white precipitate was then removed via filtration and the filtrate was concentrated via rotary evaporation. To the black oil

was added a solution of potassium hydroxide (85%, 4.042 g, 60.36 mmol) in methanol (360 mL) and the reaction mixture was refluxed for 24 hours. The reaction mixture was concentrated via rotary evaporator and the desired product was isolated via column chromatography (silica, 99% dichloromethane/ 1% methanol → 80% dichloromethane/ 20% methanol), yielding a brown oil (2.076 g, 3.473 mmol, 14.55%). Upon scale up, column chromatography conditions were adjusted (silica, isocratic 80% ethyl acetate/ 20% methanol), and yield was increased to 20%. Thin layer chromatography R_f = 0.47 (20% MeOH/DCM). ^{1}H NMR (500 MHz, $CDCl_3$) δ 8.28 (s, 2H), 4.42 – 4.26 (m, 4H), 3.35 – 3.24 (m, 4H), 3.16-3.05 (m, J = 15.8, 8.9 Hz, 4H), 2.98-2.89 (m, J = 8.0 Hz, 2H), 1.69 (p, J = 7.7 Hz, 2H), 1.58 – 1.44 (m, 8H), 1.40 (p, J = 7.6 Hz, 2H), 1.32 – 1.23 (m, 20H), 0.90 – 0.84 (m, 9H). ^{13}C{^{1}H} NMR (126 MHz, $CDCl_3$) δ 168.60, 168.53, 168.44, 168.19, 162.11 (q, $^2J_{CF}$ = 34.4 Hz), 116.72 (q, $^1J_{CF}$ = 293.1 Hz), 68.46, 68.39, 68.26, 68.19, 46.73, 46.68, 46.49, 46.40, 46.02, 45.74, 45.10, 39.35, 31.56, 31.54, 31.53, 31.49, 31.43, 31.35, 28.52, 28.49, 27.99, 27.32, 27.29, 26.94, 26.60, 26.49, 26.42, 26.35, 26.27, 25.89, 25.63, 25.33, 25.08, 22.55, 22.52, 22.44, 22.37, 13.99, 13.94, 13.87. ^{19}F NMR (471 MHz, $CDCl_3$): δ = -75.36 (s). HRMS direct injection positive mode $[MH]^+$ calculated for $[C_{28}H_{58}N_3O_3]^+$: 484.45, found 484.4466. Negative mode $[M]^-$ calculated for $[C_2F_3O_2]^-$: 112.99, found 112.9853. LC-MS rt 10.44 min, m/z 484.4457. Minor components rt 11.17 min, $[M^+]$ m/z 302.2319; 12.04 min, $[M^+]$ m/z 866.7633. Minor components observed in reverse phase LC-MS were not detectable in direct injection MS nor in NMR spectroscopy.

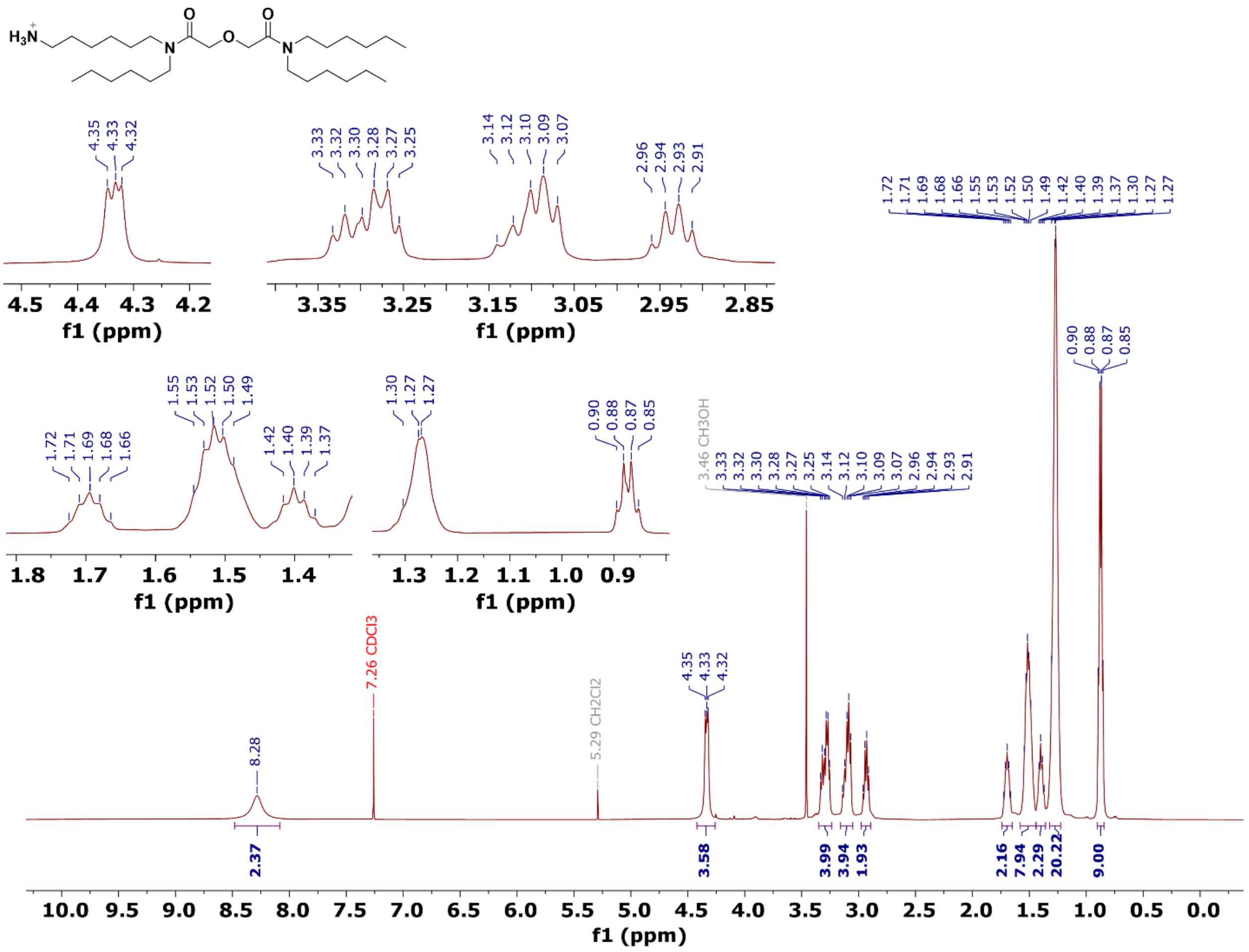

Figure S1. ^{1}H NMR of aTHDGA. 500 MHz 1H NMR of N-(6-aminohexyl)-N,N',N'-trihexyl diglycolamide trifluoroacetate in CDCl3.

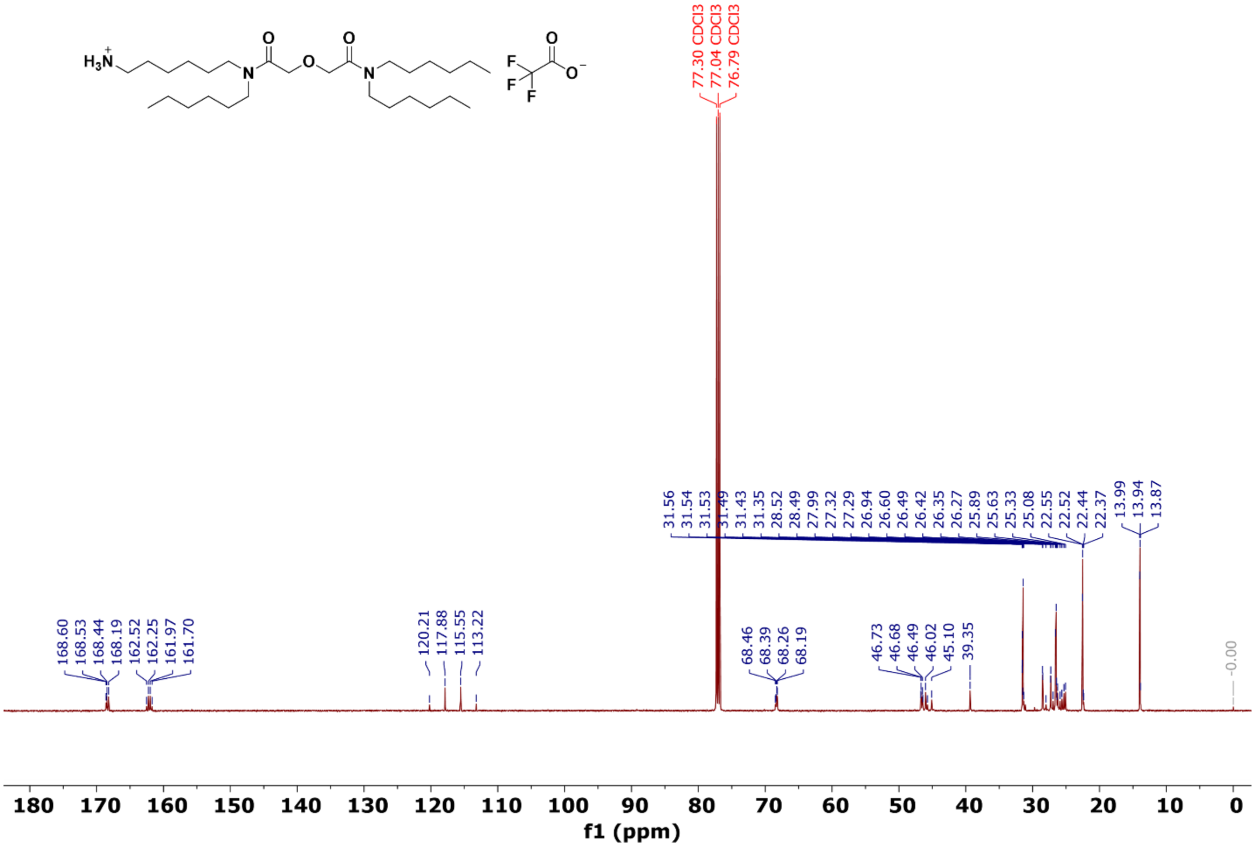

Figure S2. ^{13}C NMR of aTHDGA. 126 MHz ^{13}C NMR of *N*-(6-aminohexyl)-*N*,*N*',*N*'-trihexyl diglycolamide trifluoroacetate in $CDCl_3$.

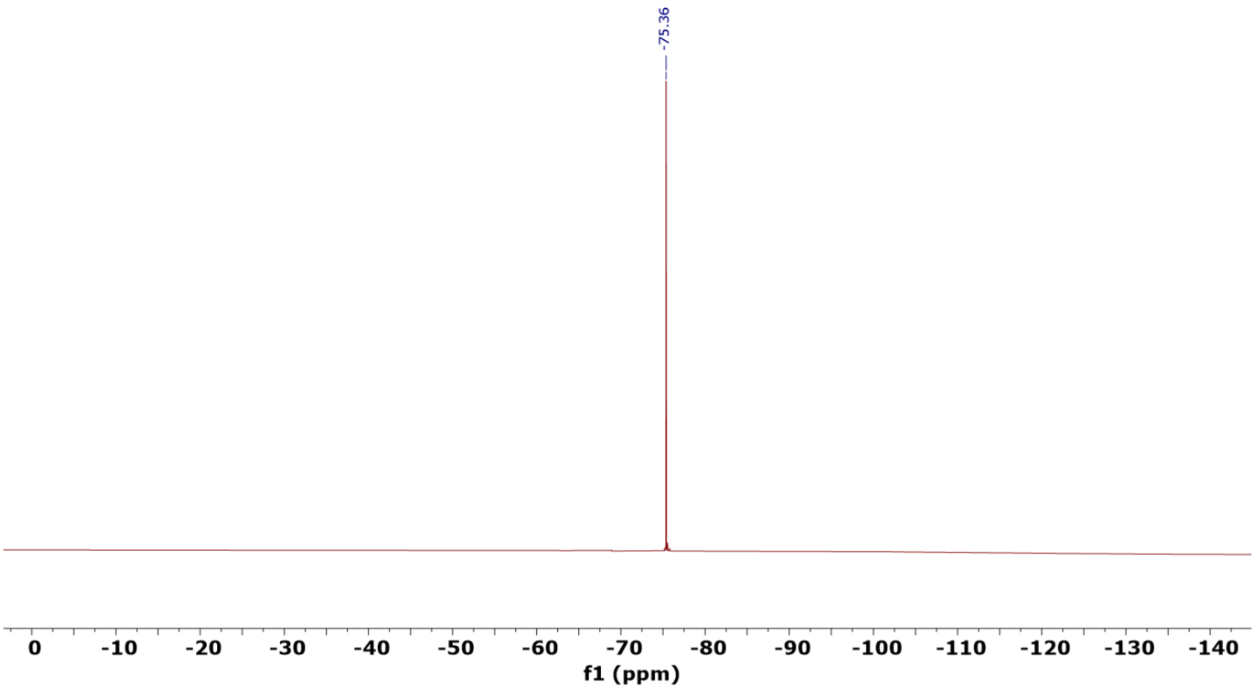

Figure S3. ^{19}F NMR of aTHDGA. 471 MHz ^{19}F NMR of *N*-(6-aminohexyl)-*N*,*N*',*N*'-trihexyl diglycolamide trifluoroacetate in $CDCl_3$.

Synthesis of THDGA membrane

N-(6-aminohexyl)-*N*,*N'*,*N'*-trihexyl diglycolamide trifluoroacetate (0.483 g, 0.808 mmol) was dissolved in ethanol (40 mL). Then, cesium carbonate (0.330 g, 1.01 mmol) and potassium iodide (0.178 g, 1.07 mmol) were added to the solution and stirred on a stir table to mix the reaction solution. A 44 mm diameter PVBC membrane (100 mg PVBC) on P4 filter paper support was submerged, membrane-side down, in the reaction solution. The membrane-containing reaction solution was refluxed in a sand bath for 48 hours. After the reaction, the membrane was removed from the solution, submerged and shaken (LAB-LINE 3528−5 Orbit Environ-Shaker table) in 40 mL of ethanol for 15 minutes, submerged and shaken in 40 mL of water for 15 minutes, submerged and shaken in 40 mL of fresh water for 15 minutes, and finally submerged and shaken in 40 mL of ethanol for 15 minutes. The membrane was dried in the fume hood.

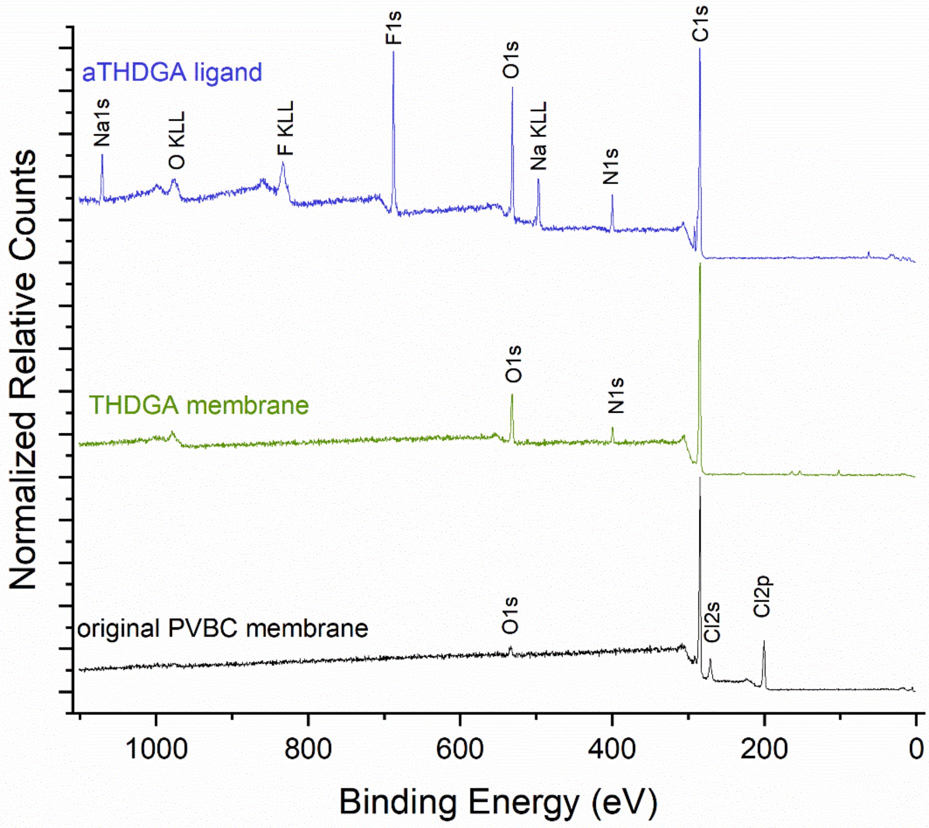

Figure S4. X-ray photoelectron spectra (resolution 0.4 eV energy step) of PVBC membrane, THDGA membrane, aTHDGA ligand. The THDGA membrane is free of major contaminating ions (e,g., Cs, I, K, F used in the reagents or present in the counterions for the reaction) and the Cl from the original PVBC has been completely consumed at the membrane surface.

Resin and Membrane Morphology

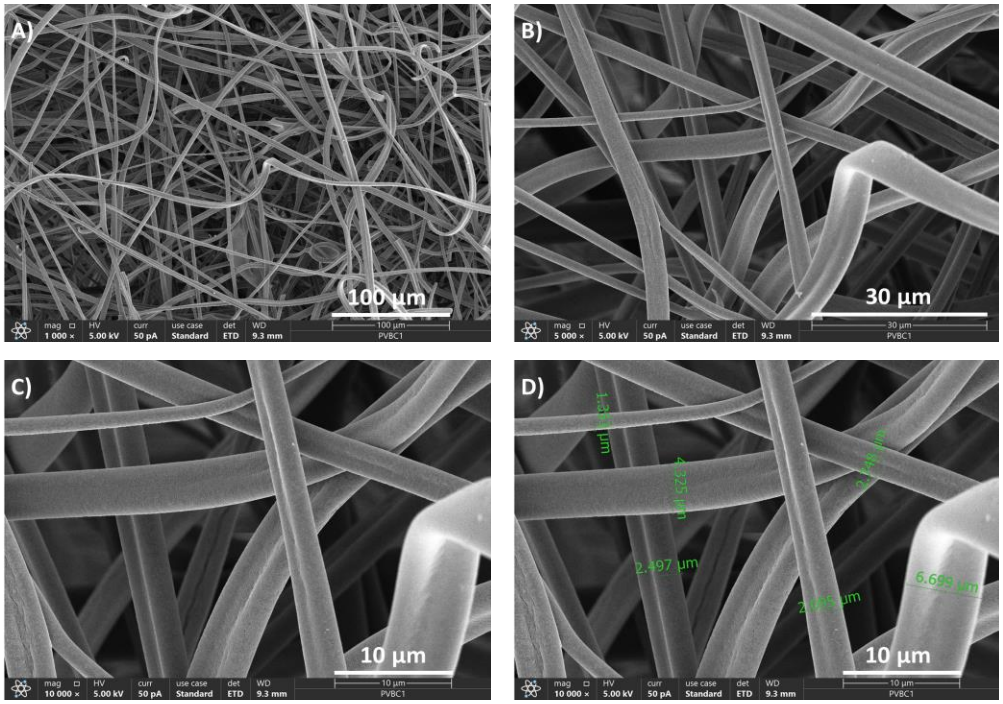

Figure S5. (A-C) SEM images of a PVBC membrane at different magnifications, and D) fiber diameter analysis of a PVBC membrane. The mean fiber diameter for n = 6 is 3.3 ± 1.7 µm.

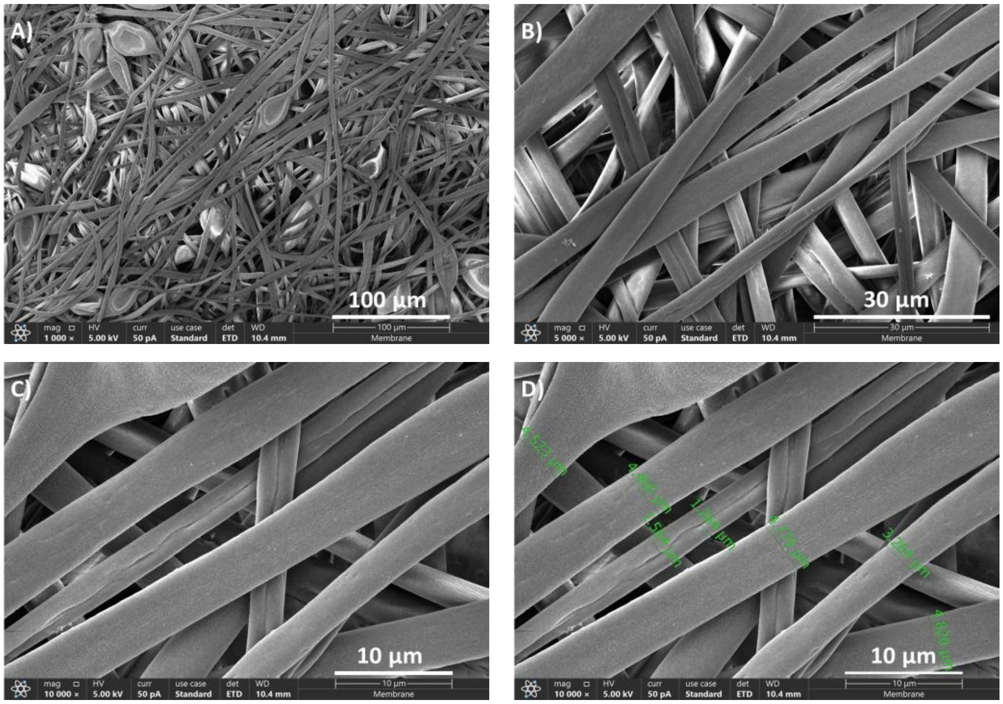

Figure S6. (A-C) SEM images of a THDGA membrane at different magnifications, and D) fiber diameter analysis of a THDGA membrane. The mean fiber diameter for n = 7 is 3.2 ± 1.5 µm.

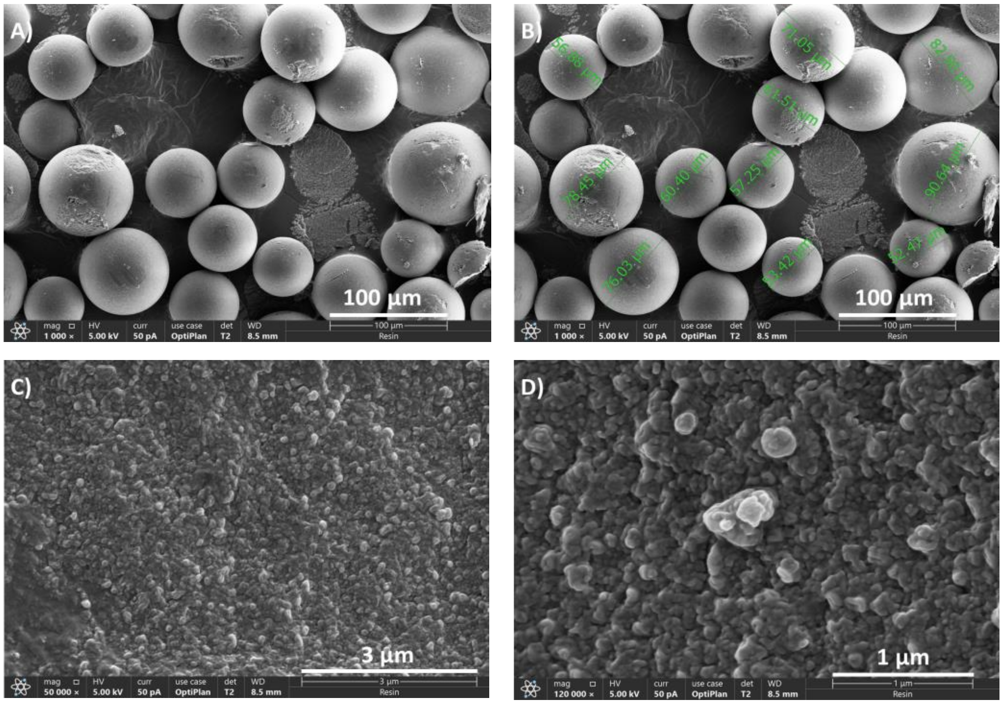

Figure S7. A) SEM image of TODGA resins, B) particle size analysis measuring the diameter of the particles, C) TODGA resin surface, and D) TODGA resin surface at increased magnification. The mean particle size for n=11 is 67 ± 13 µm.

Porosity Measurements

Table S1. Porosity measurements for THDGA membrane with fiber diameter data and its corresponding calculated surface area.

Dry weight (g)	Wet weight (g)	Porosity	Average porosity	Standard deviation	Fiber diameter * 10^{-6} (m)	Surface area (m^2/mL)
0.0019	0.0092	0.84	0.80	0.08	3.2 ± 1.5	0.25
0.0017	0.0047	0.71				
0.0019	0.0099	0.85				

Table S2. Porosity measurements for PVBC membrane with fiber diameter data and its corresponding calculated surface area.

Dry weight (g)	Wet weight (g)	Porosity	Average porosity	Standard deviation	Fiber diameter * 10^{-6} (m)	Surface area (m^2/mL)
0.0016	0.0043	0.70	0.68	0.057	3.3 ± 1.7	0.39
0.002	0.0043	0.61				
0.0015	0.0043	0.72				

Permeance Measurements

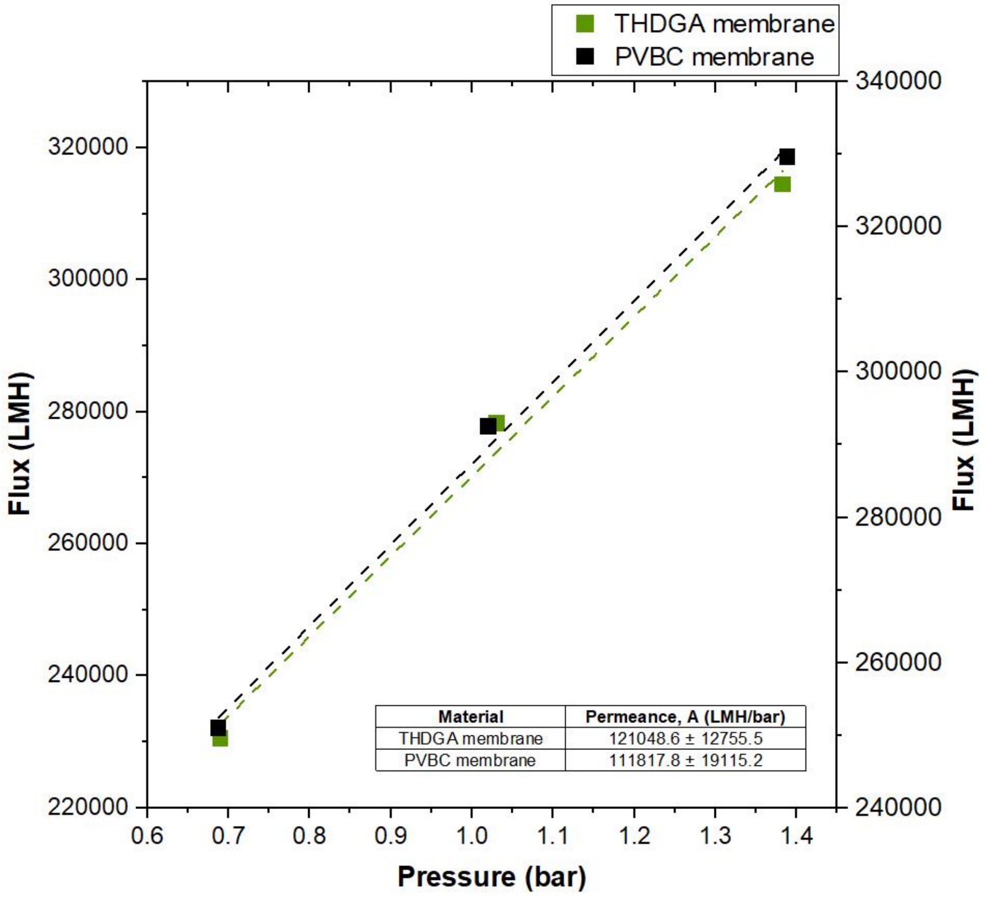

Figure S8. Flux versus pressure plots to determine the permeance values of THDGA and PVBC membranes. The permeance values are calculated from the slopes of the linear fits.

Adsorption Isotherms

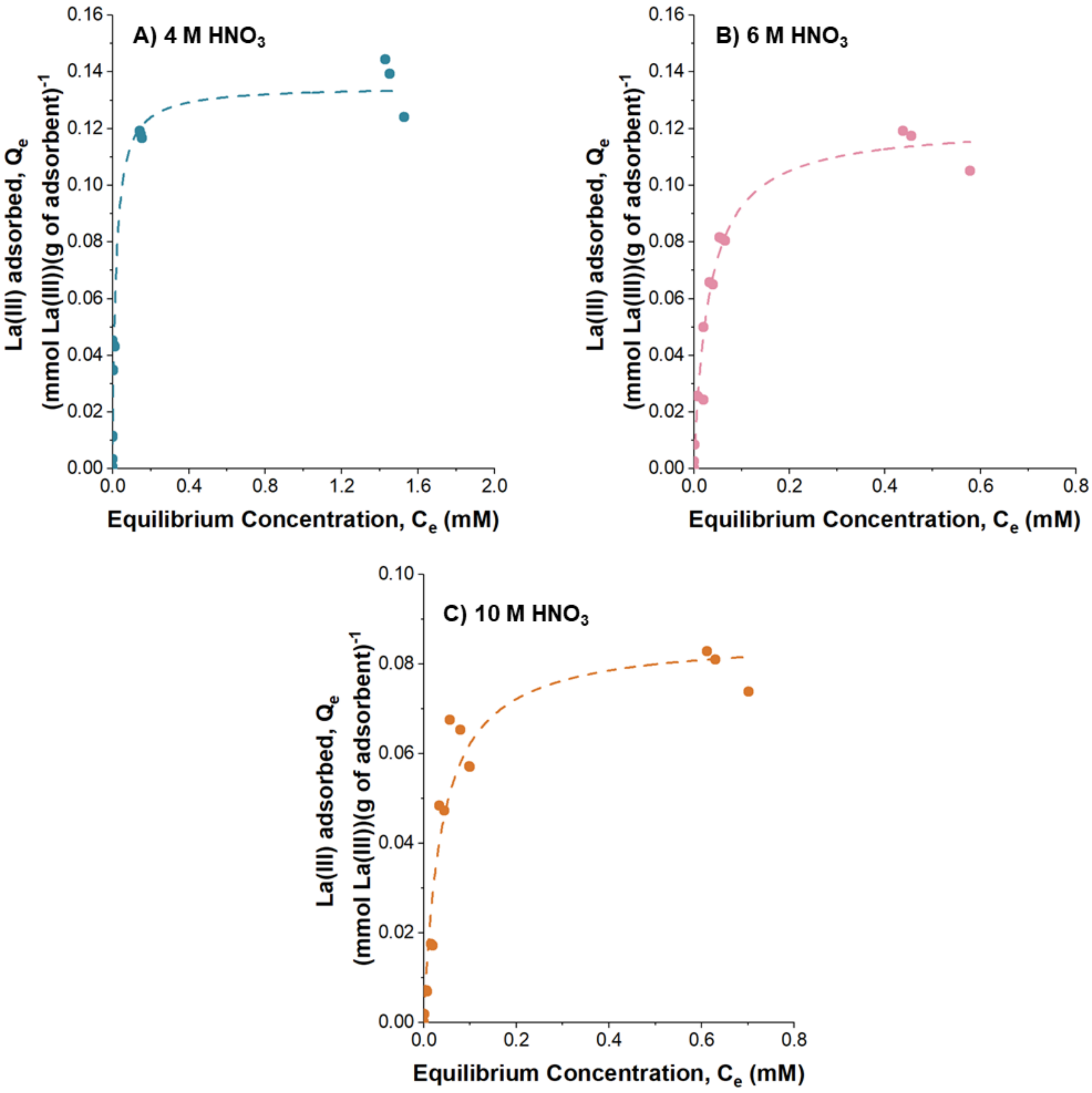

Figure S9. Equilibrium adsorption plots for La(III) in TODGA resins at A) 4 M nitric acid, B) 6 M nitric acid, C) 10 M nitric acid using the Langmuir isotherm model. The dots represent the experimental data and the dashed curves represent the isotherm model.

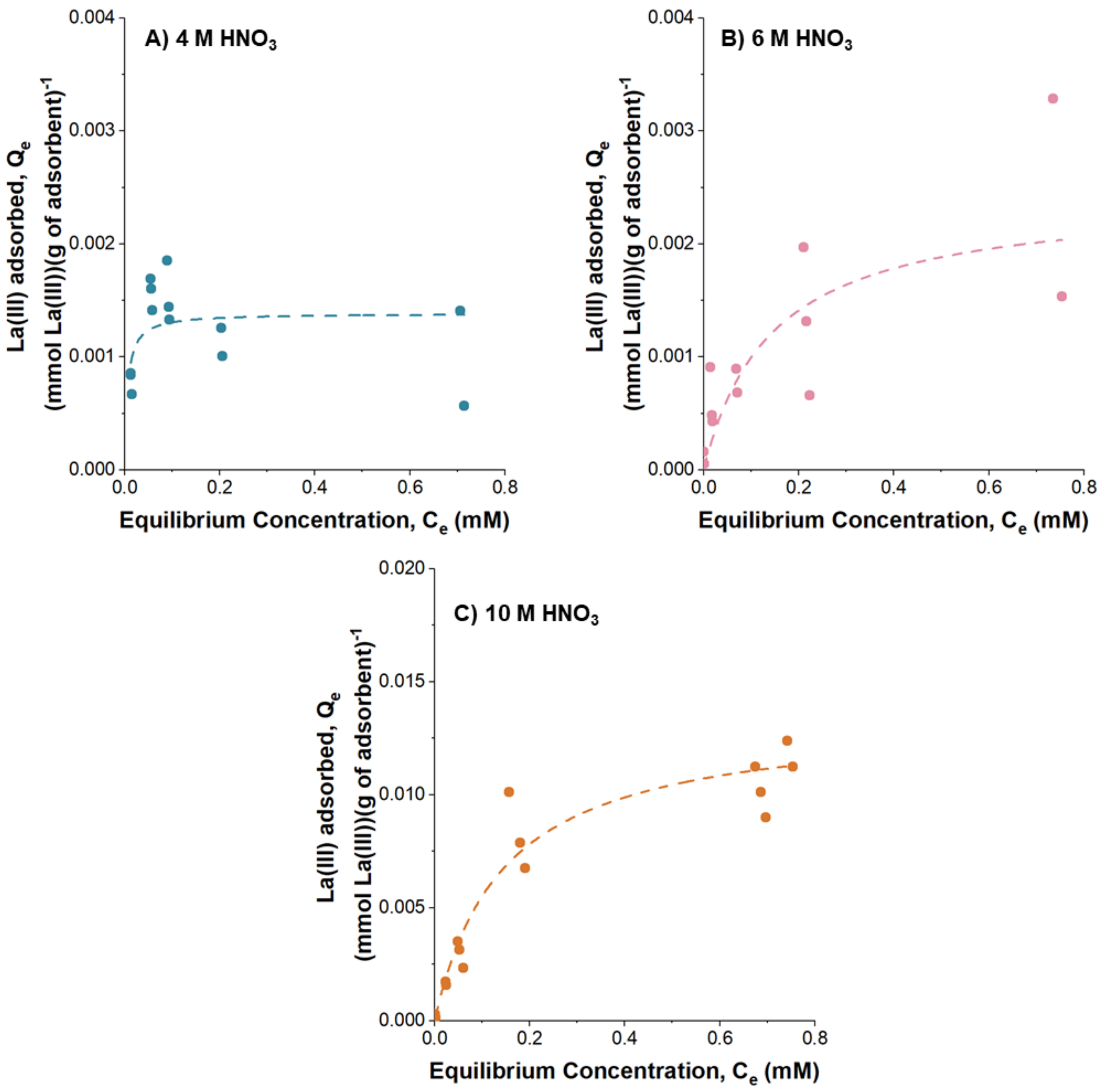

Figure S10. Equilibrium adsorption plots for La(III) on THDGA membranes at A) 4 M nitric acid, B) 6 M nitric acid, and C) 10 M nitric acid using the Langmuir isotherm model. The dots represent the experimental data and the dashed curves represent the isotherm model.

Table S3. Langmuir fitting parameters for La(III) adsorbed in TODGA resins and THDGA membranes.

Nitric Acid Concentration	TODGA resins		THDGA membranes	
	Q_{max} (mmol/g)	K_L (mM^{-1})	Q_{max} (mmol/g)	K_L (mM^{-1})
4 M	0.13 ± 0.006	57.64 ± 12.64	0.003 ± 4.07 × 10^{-4}	38.4 ± 17.60
6 M	0.12 ± 0.004	31.88 ± 3.20	0.002 ± 5.43 × 10^{-4}	7.0 ± 4.67
10 M	0.086 ± 0.004	26.06 ± 4.16	0.013 ± 9.30 × 10^{-4}	6.94 ± 1.63

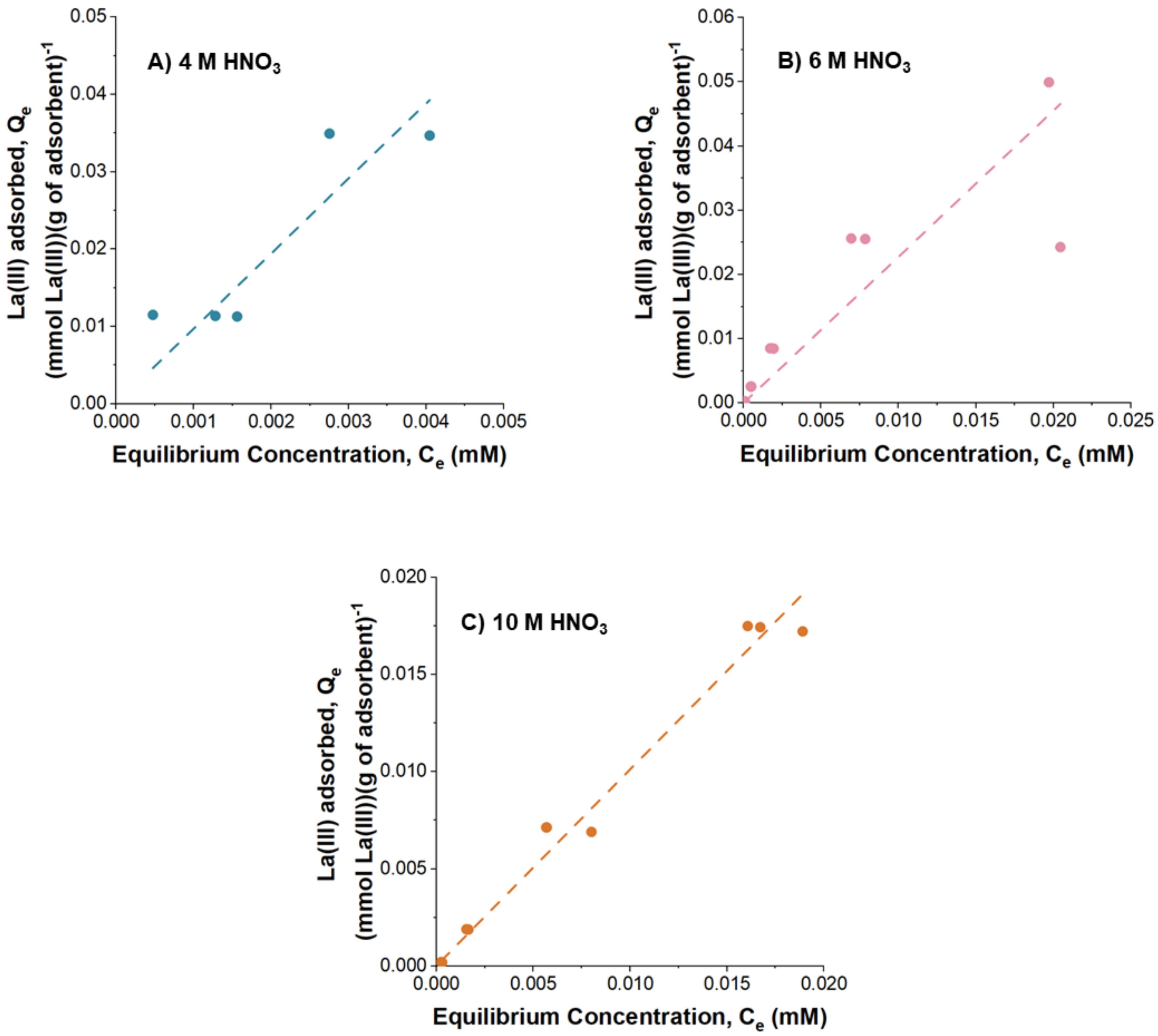

Figure S11. Linear isotherm plots for La(III) adsorbed to TODGA resins for low concentration regions of the equilibrium adsorption measurements at A) 4 M nitric acid, B) 6 M nitric acid, and C) 10 M nitric acid. The dots represent the experimental data and the dashed lines represent the isotherm model.

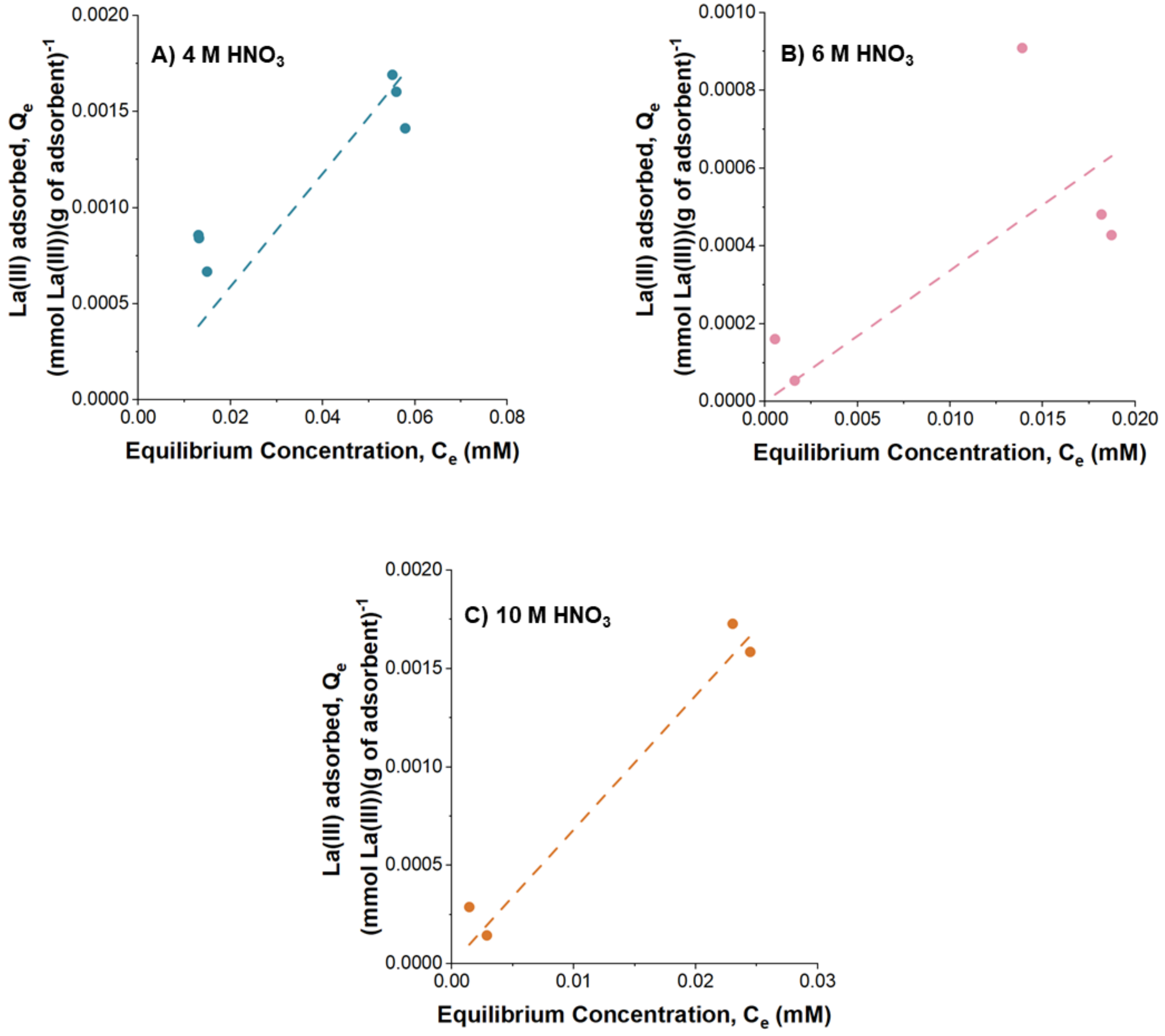

Figure S12. Linear isotherm plots for La(III) adsorbed to THDGA membranes for low concentration regions of the equilibrium adsorption measurements at A) 4 M nitric acid, B) 6 M nitric acid, and C) 10 M nitric acid. The dots represent the experimental data, and the dashed lines represent the isotherm model.

Table S4. Linear fitting parameters for La(III) adsorbed to TODGA resins and THDGA membranes for the linear regions of the equilibrium adsorption data.

Nitric Acid Concentration	TODGA resins	THDGA membranes
	K (mL/g)	K (mL/g)
4 M	9,710 ± 1,160	39.4 ± 3.4
6 M	2,270 ± 180	33.7 ± 7.9
10 M	1,010 ± 30	68.1 ± 3.6

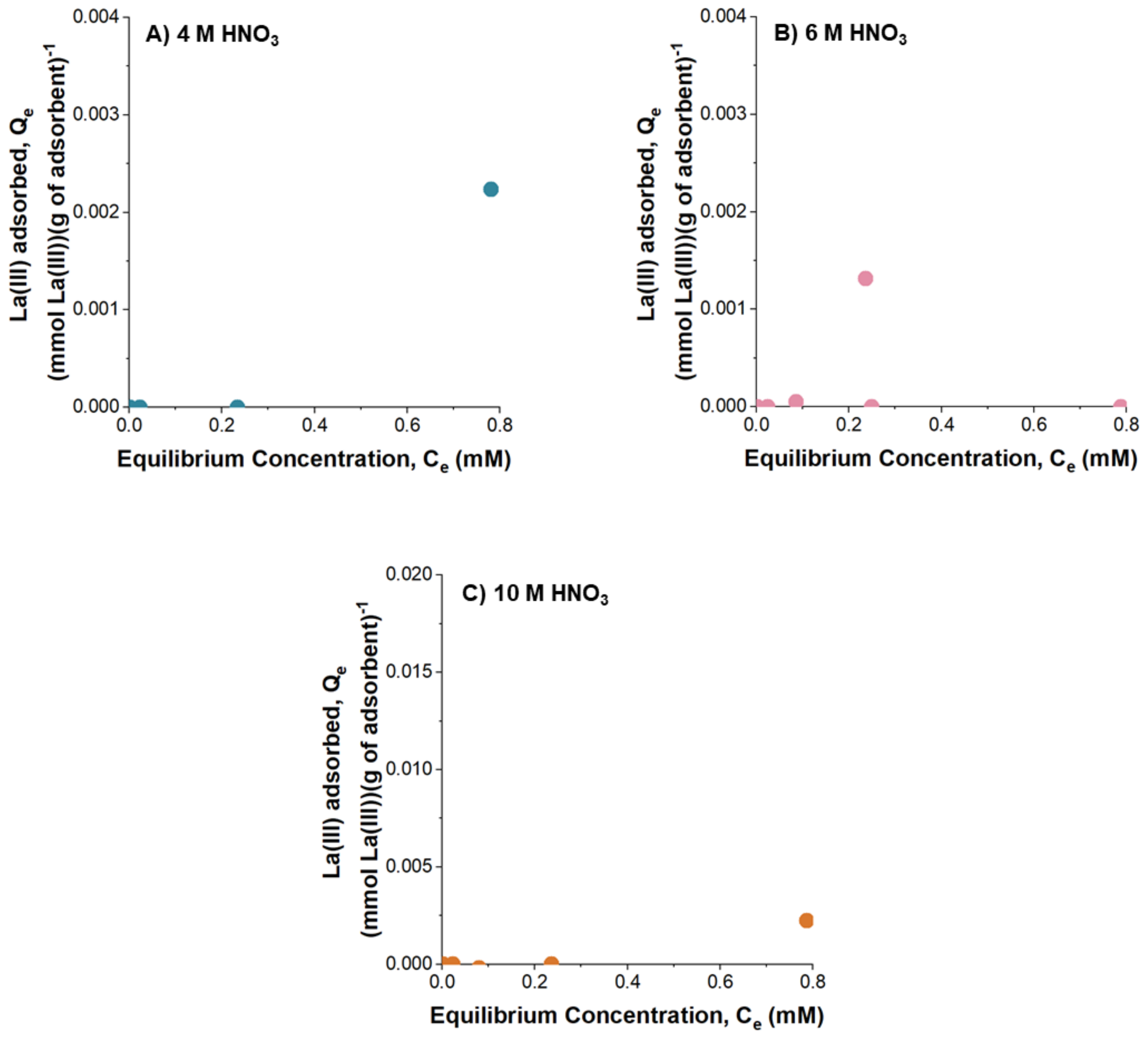

Figure S13. Equilibrium adsorption control plot for La(III) in PVBC membrane at A) 4 M nitric acid, B) 6 M nitric acid, C) 10 M nitric acid. The dots represent the experimental data.

Lanthanum desorption from THDGA membranes

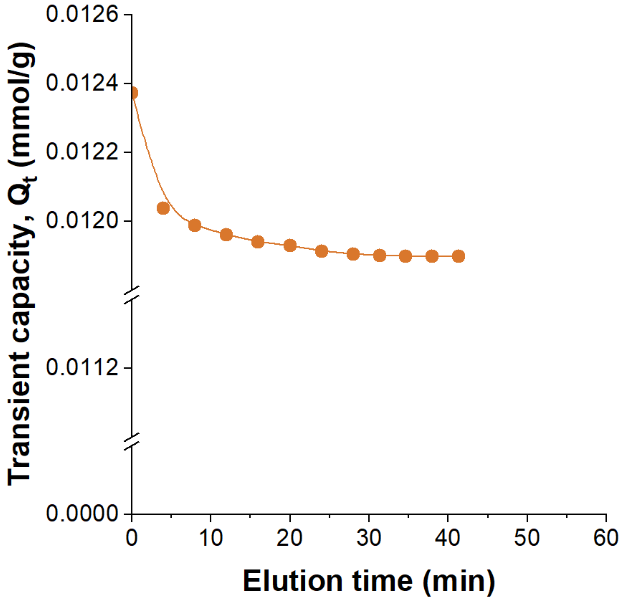

Figure S14. La(III) desorption from THDGA membrane using pH 2 nitric acid. The initial La(III) loaded on the fibers was 0.0124 mmol/g. Elution was performed for 1 hour after which the La(III) concentration in the eluent fractions was below the detection limits of the ICP-OES.

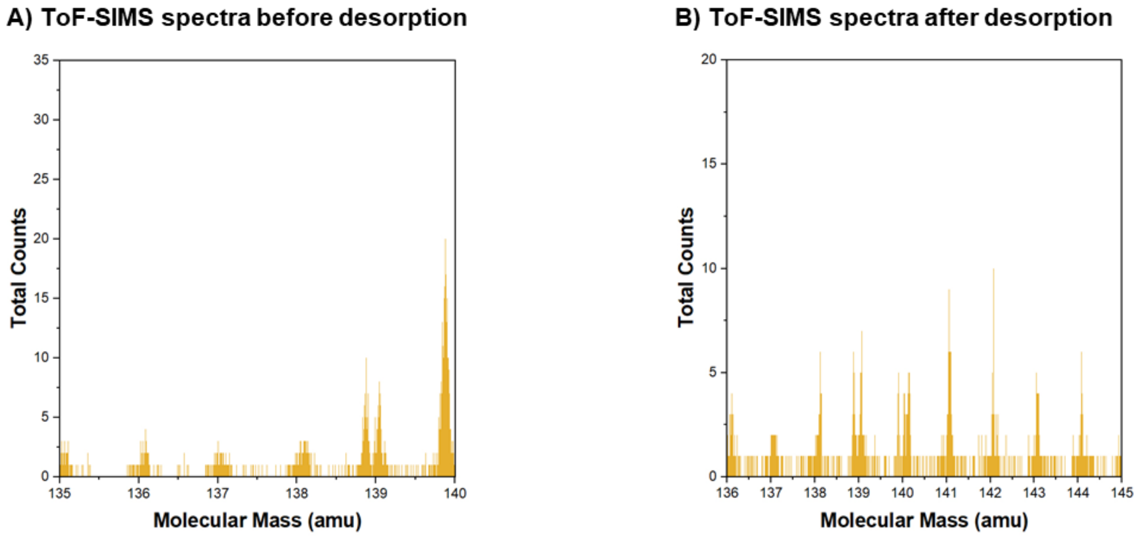

Figure S15. ToF-SIMS spectra of THDGA membrane A) before attempting elution with pH 2 nitric acid and B) after elution with pH 2 nitric acid. The theoretical atomic mass of Lanthanum is 138.9057 amu.

One-way ANOVA: Membrane KL versus Acid Strength

Method

Null hypothesis	All means are equal
Alternative hypothesis	Not all means are equal
Significance level	α = 0.05

Equal variances were assumed for the analysis.

Factor Information

Factor	Levels	Values
Acid	3	4, 6, 10

Analysis of Variance

Source	DF	Adj SS	Adj MS	F-Value	P-Value
Acid	2	6444.3	3222.2	30.07	0.001
Error	6	642.9	107.1		
Total	8	7087.2			

Model Summary

S	R-sq	R-sq(adj)	R-sq(pred)
10.3512	90.93%	87.91%	79.59%

Means

Acid	N	Mean	StDev	95% CI
4	3	64.13	16.19	(49.51, 78.75)
6	3	7.85	7.09	(-6.78, 22.47)
10	3	6.90	3.04	(-7.73, 21.52)

Pooled StDev = 10.3512

Tukey Pairwise Comparisons

Grouping Information Using the Tukey Method and 95% Confidence

Acid	N	Mean	Grouping	
4	3	64.13	A	
6	3	7.85		B
10	3	6.90		B

Means that do not share a letter are significantly different.

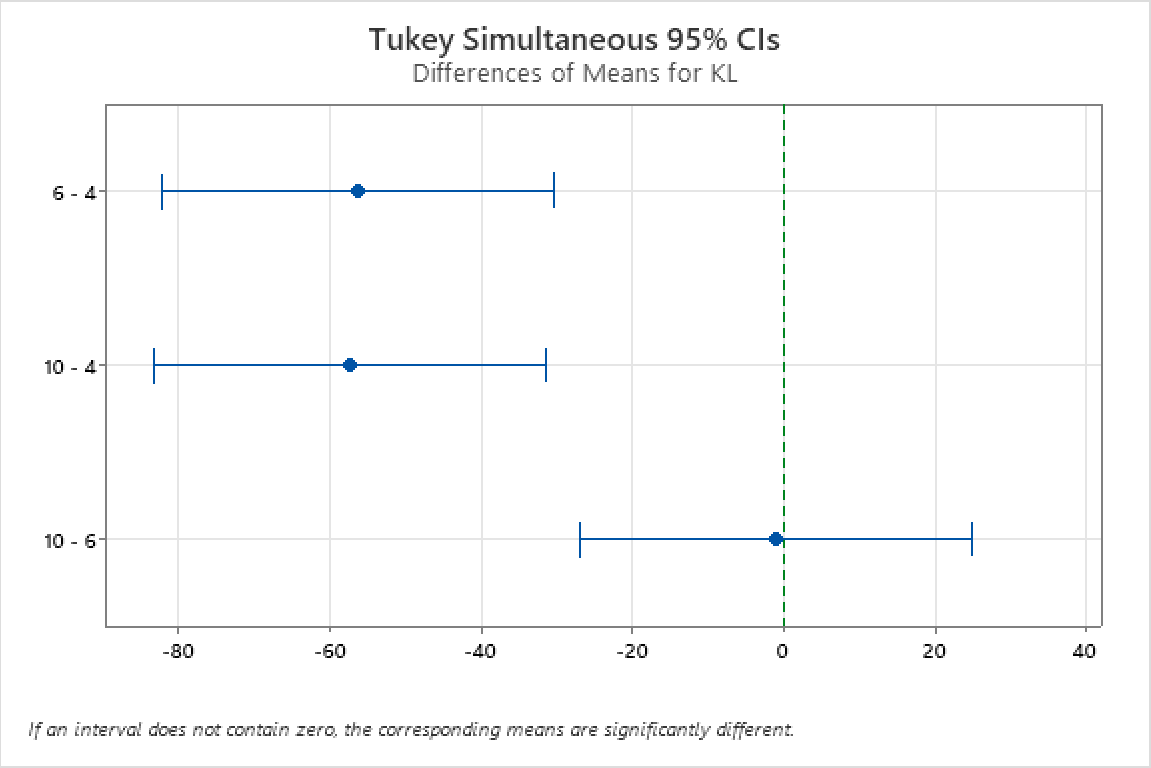
Tukey Simultaneous 95% CIs
Differences of Means for KL
6 - 4
10 - 4
10 - 6
-80
-60
-40
-20
0
20
40
If an interval does not contain zero, the corresponding means are significantly different.

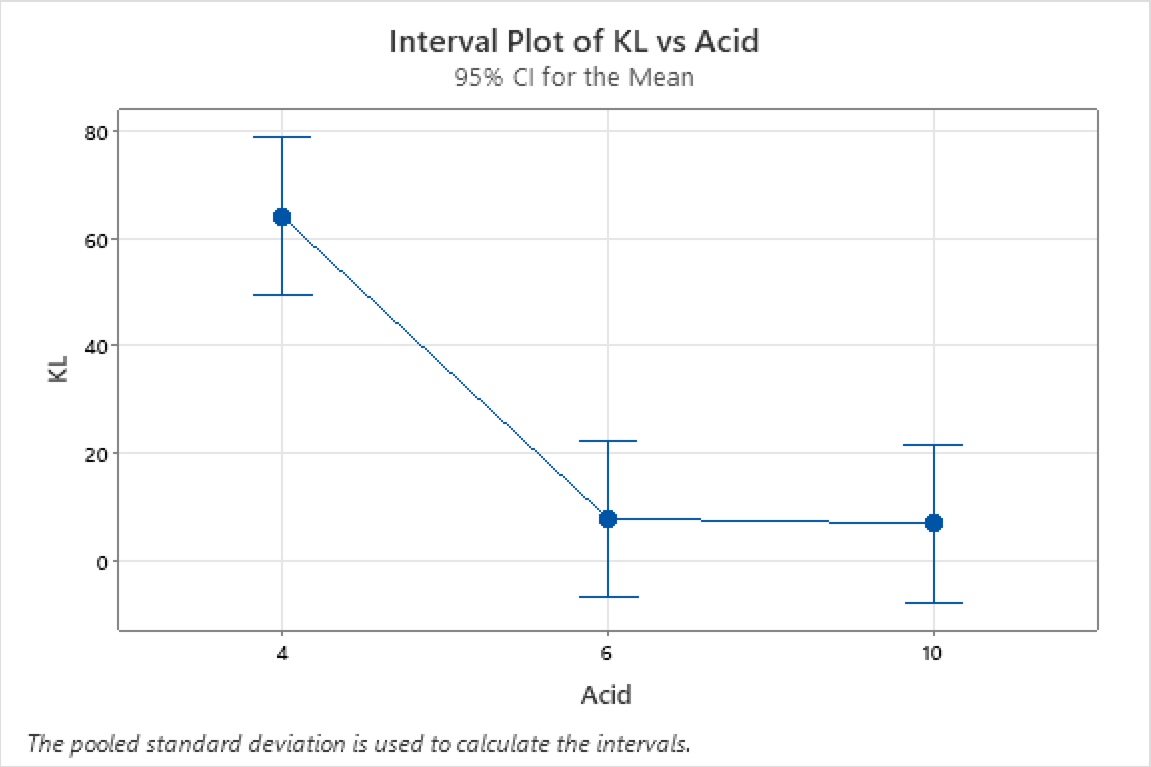
Interval Plot of KL vs Acid
95% CI for the Mean
80
60
40
20
0
KL
4
6
10
Acid
The pooled standard deviation is used to calculate the intervals.

One-way ANOVA: Membrane Qmax versus Acid Strength

Method

Null hypothesis	All means are equal
Alternative hypothesis	Not all means are equal
Significance level	α = 0.05

Equal variances were assumed for the analysis.

Factor Information

Factor	Levels	Values
Acid	3	4, 6, 10

Analysis of Variance

Source	DF	Adj SS	Adj MS	F-Value	P-Value
Acid	2	0.000262	0.000131	50.23	0.000
Error	6	0.000016	0.000003		
Total	8	0.000278			

Model Summary

S	R-sq	R-sq(adj)	R-sq(pred)
0.0016151	94.36%	92.49%	87.32%

Means

Acid	N	Mean	StDev	95% CI
4	3	0.001870	0.000426	(-0.000412, 0.004152)
6	3	0.00306	0.00216	(0.00078, 0.00535)
10	3	0.013867	0.001720	(0.011585, 0.016148)

Pooled StDev = 0.00161510

Tukey Pairwise Comparisons

Grouping Information Using the Tukey Method and 95% Confidence

Acid	N	Mean	Grouping	
10	3	0.013867	A	
6	3	0.00306		B
4	3	0.001870		B

Means that do not share a letter are significantly different.

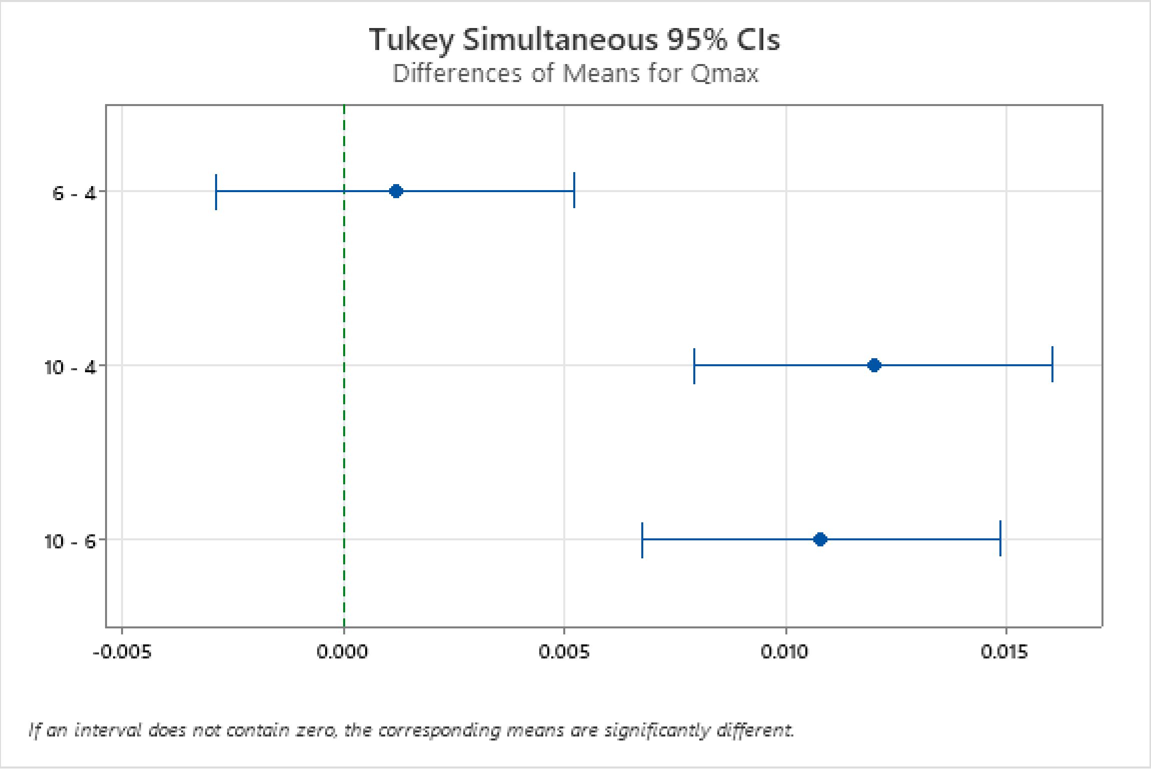
Tukey Simultaneous 95% CIs
Differences of Means for Qmax
6 - 4
10 - 4
10 - 6
-0.005
0.000
0.005
0.010
0.015
If an interval does not contain zero, the corresponding means are significantly different.

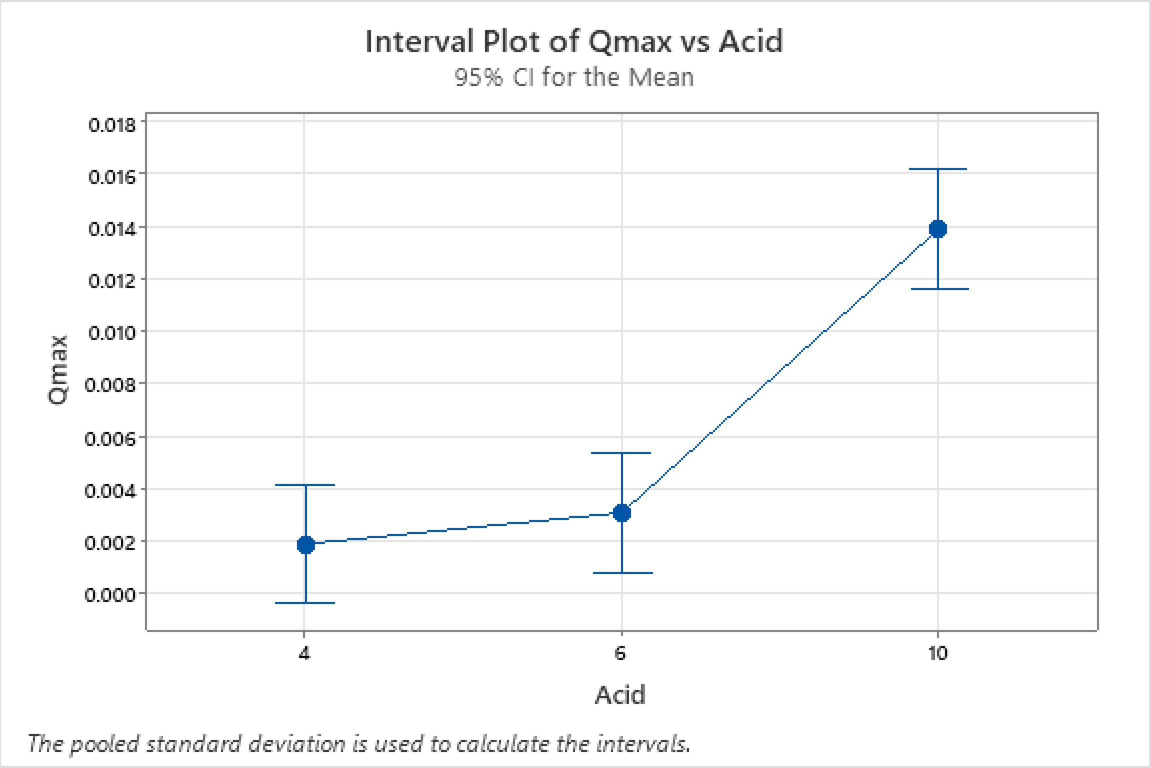
Interval Plot of Qmax vs Acid
95% CI for the Mean
Qmax
0.018
0.016
0.014
0.012
0.010
0.008
0.006
0.004
0.002
0.000
4
6
10
Acid
The pooled standard deviation is used to calculate the intervals.

One-way ANOVA: Resin KL versus Acid Strength

Method

Null hypothesis	All means are equal
Alternative hypothesis	Not all means are equal
Significance level	α = 0.05

Equal variances were assumed for the analysis.

Factor Information

Factor	Levels	Values
Acid	3	4, 6, 10

Analysis of Variance

Source	DF	Adj SS	Adj MS	F-Value	P-Value
Acid	2	835.8	417.92	10.68	0.011
Error	6	234.7	39.12		
Total	8	1070.6			

Model Summary

S	R-sq	R-sq(adj)	R-sq(pred)
6.25483	78.07%	70.76%	50.67%

Means

Acid	N	Mean	StDev	95% CI
4	3	48.99	4.48	(40.15, 57.83)
6	3	33.09	9.75	(24.25, 41.92)
10	3	25.931	1.488	(17.095, 34.767)

Pooled StDev = 6.25483

Tukey Pairwise Comparisons

Grouping Information Using the Tukey Method and 95% Confidence

Acid	N	Mean	Grouping	
4	3	48.99	A	
6	3	33.09		B
10	3	25.931		B

Means that do not share a letter are significantly different.

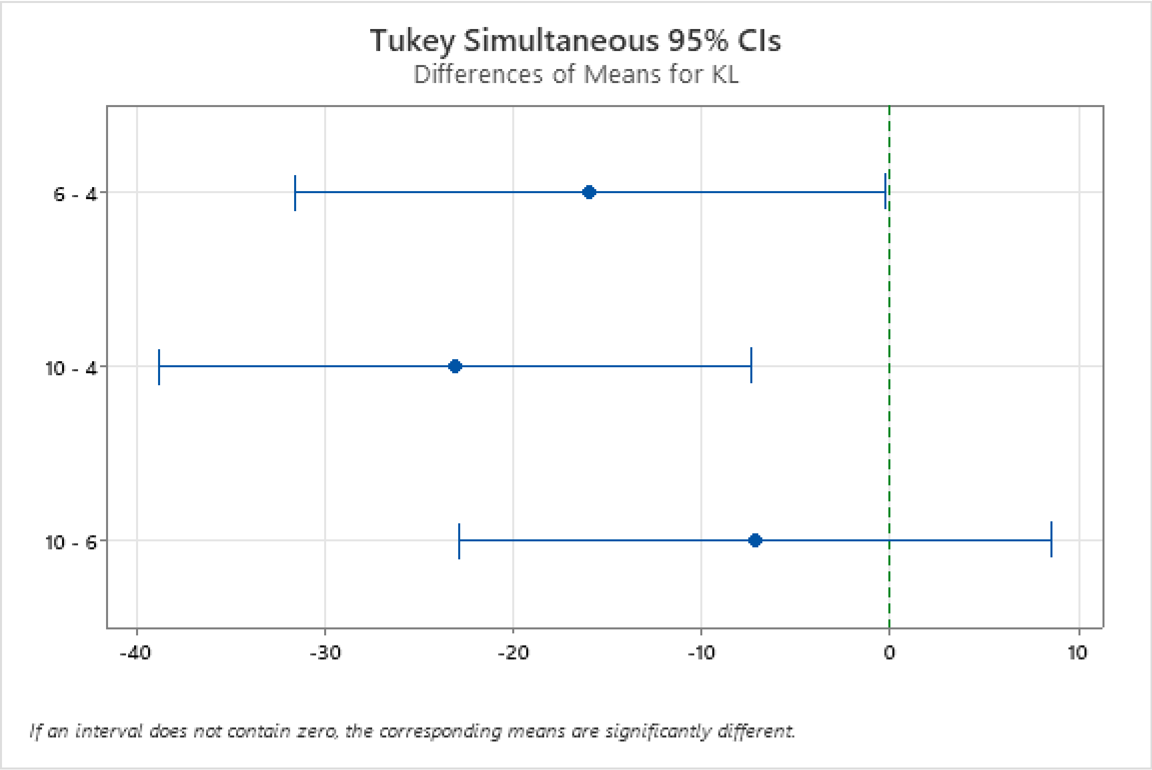
Tukey Simultaneous 95% CIs
Differences of Means for KL
6 - 4
10 - 4
10 - 6
-40
-30
-20
-10
0
10
If an interval does not contain zero, the corresponding means are significantly different.

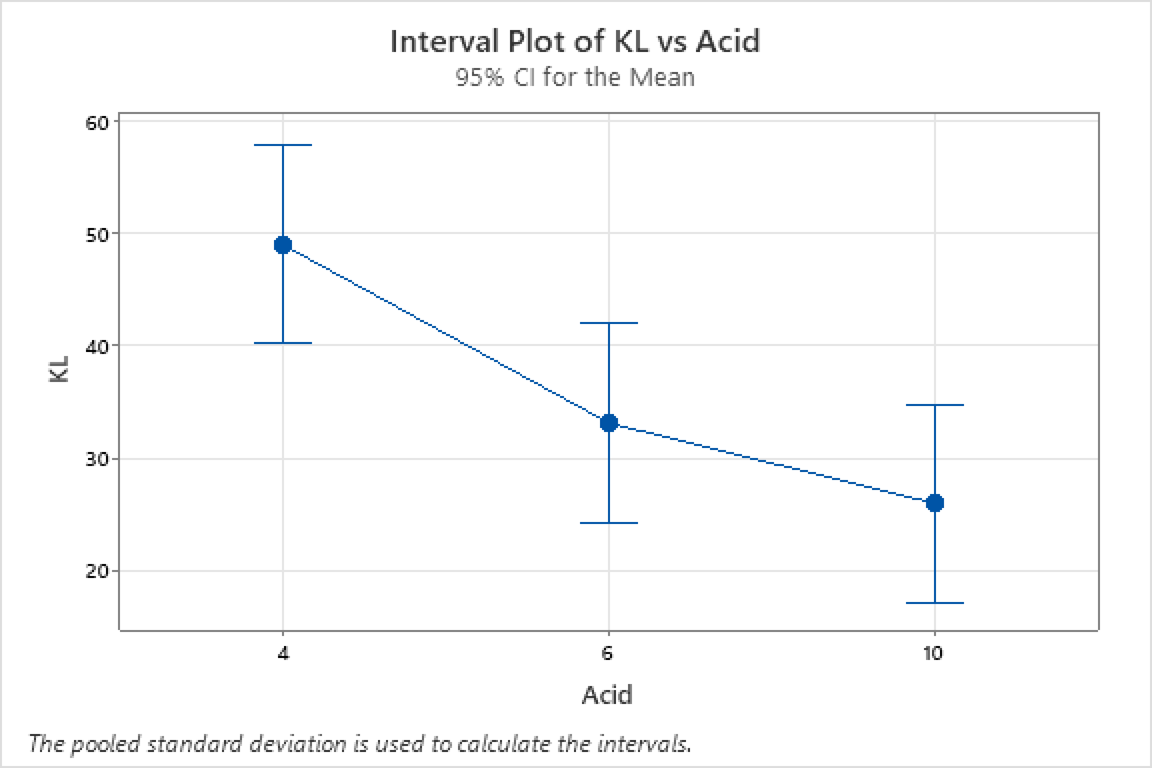
Interval Plot of KL vs Acid
95% CI for the Mean
KL
60
50
40
30
20
4
6
10
Acid
The pooled standard deviation is used to calculate the intervals.

One-way ANOVA: Resin Qmax versus Acid Strength

Method

Null hypothesis	All means are equal
Alternative hypothesis	Not all means are equal
Significance level	α = 0.05

Equal variances were assumed for the analysis.

Factor Information

Factor	Levels	Values
Acid	3	4, 6, 10

Analysis of Variance

Source	DF	Adj SS	Adj MS	F-Value	P-Value
Acid	2	0.003895	0.001948	36.82	0.000
Error	6	0.000317	0.000053		
Total	8	0.004212			

Model Summary

S	R-sq	R-sq(adj)	R-sq(pred)
0.0072723	92.47%	89.96%	83.05%

Means

Acid	N	Mean	StDev	95% CI
4	3	0.13557	0.00585	(0.12529, 0.14584)
6	3	0.12233	0.01026	(0.11206, 0.13261)
10	3	0.08633	0.00437	(0.07606, 0.09661)

Pooled StDev = 0.00727232

Tukey Pairwise Comparisons

Grouping Information Using the Tukey Method and 95% Confidence

Acid	N	Mean	Grouping	
4	3	0.13557	A	
6	3	0.12233	A	
10	3	0.08633		B

Means that do not share a letter are significantly different.

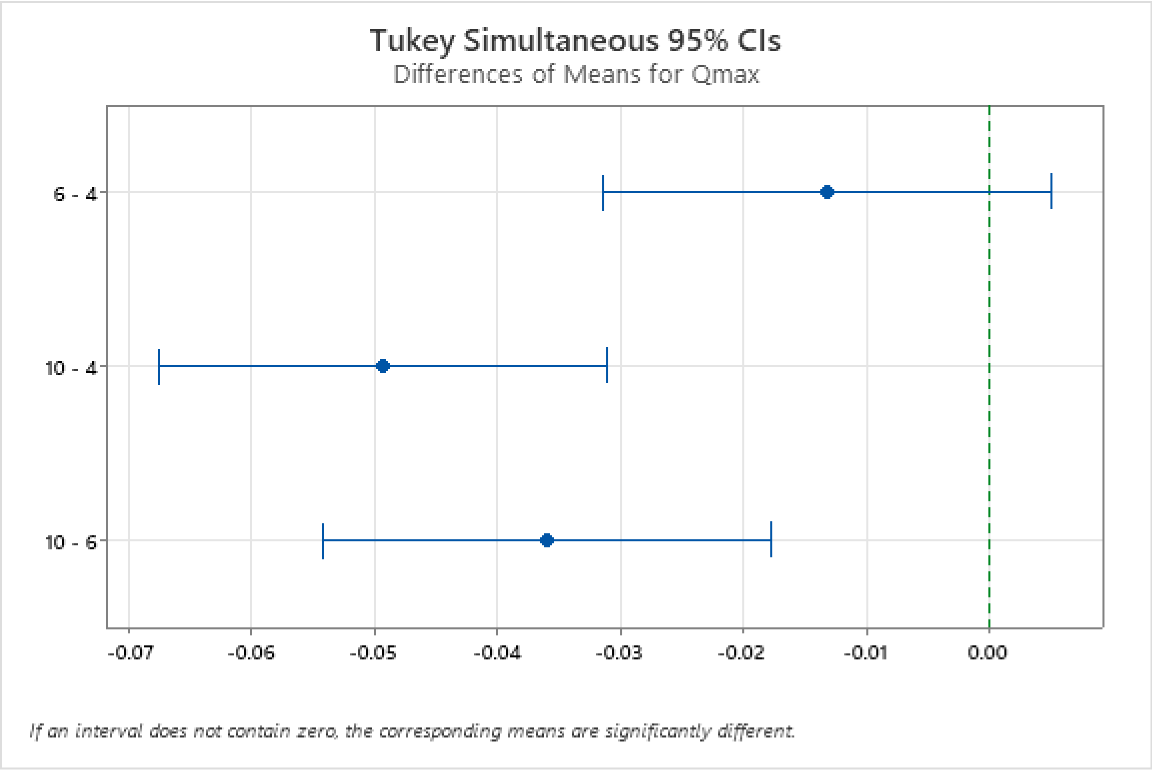
Tukey Simultaneous 95% CIs
Differences of Means for Qmax
6 - 4
10 - 4
10 - 6
-0.07
-0.06
-0.05
-0.04
-0.03
-0.02
-0.01
0.00
If an interval does not contain zero, the corresponding means are significantly different.

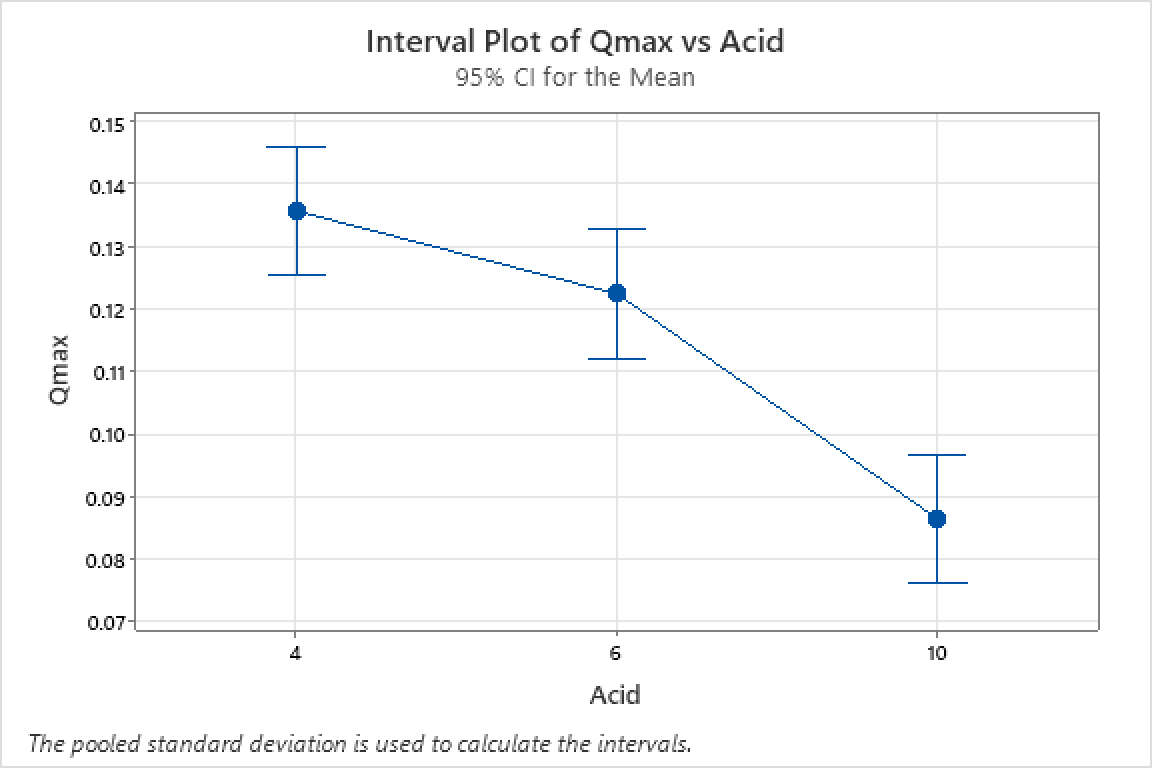
Interval Plot of Qmax vs Acid
95% CI for the Mean
Qmax
0.15
0.14
0.13
0.12
0.11
0.10
0.09
0.08
0.07
4
6
10
Acid
The pooled standard deviation is used to calculate the intervals.

One-way ANOVA: 4M Acid KL versus Support

(1 = membrane, 2= resin)

Method

Null hypothesis	All means are equal
Alternative hypothesis	Not all means are equal
Significance level	α = 0.05

Equal variances were assumed for the analysis.

Factor Information

Factor	Levels	Values
Support	2	1, 2

Analysis of Variance

Source	DF	Adj SS	Adj MS	F-Value	P-Value
Support	1	343.8	343.8	2.44	0.193
Error	4	564.1	141.0		
Total	5	907.9			

Model Summary

S	R-sq	R-sq(adj)	R-sq(pred)
11.8755	37.87%	22.34%	0.00%

Means

Support	N	Mean	StDev	95% CI
1	3	64.13	16.19	(45.09, 83.17)
2	3	48.99	4.48	(29.95, 68.03)

Pooled StDev = 11.8755

Tukey Pairwise Comparisons

Grouping Information Using the Tukey Method and 95% Confidence

Support	N	Mean	Grouping
1	3	64.13	A
2	3	48.99	A

Means that do not share a letter are significantly different.

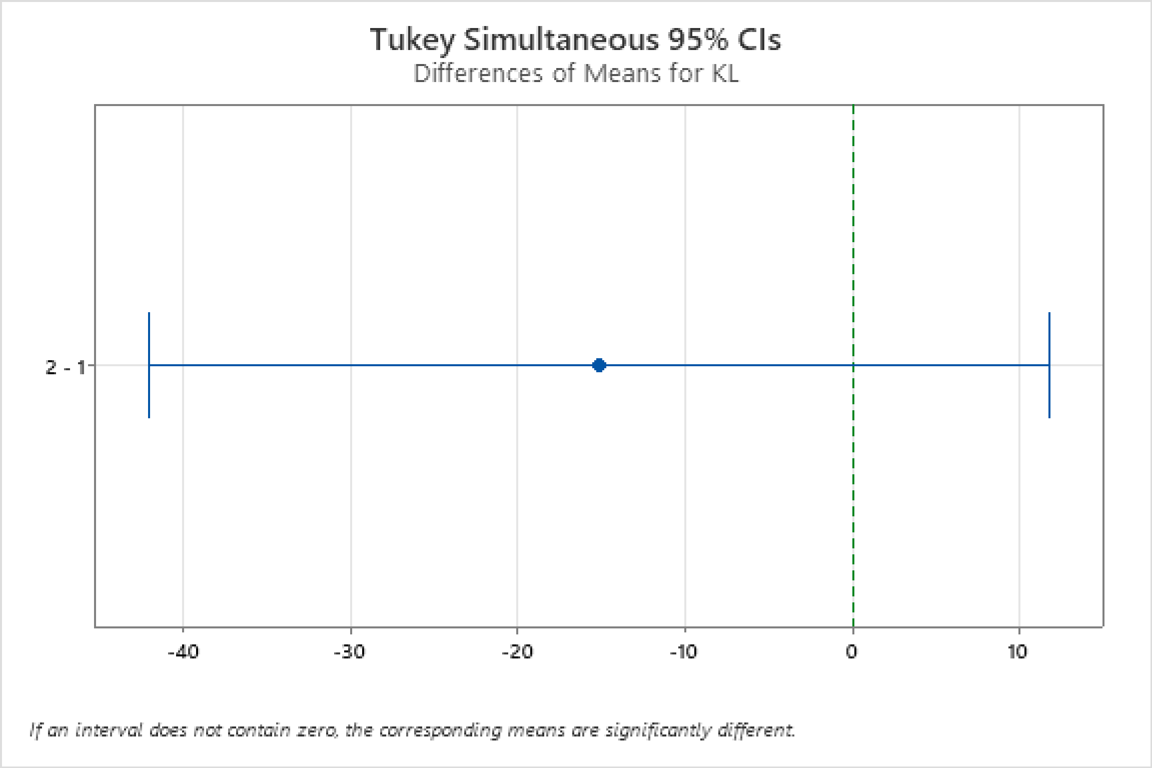
Tukey Simultaneous 95% CIs
Differences of Means for KL
2 - 1
-40
-30
-20
-10
0
10
If an interval does not contain zero, the corresponding means are significantly different.

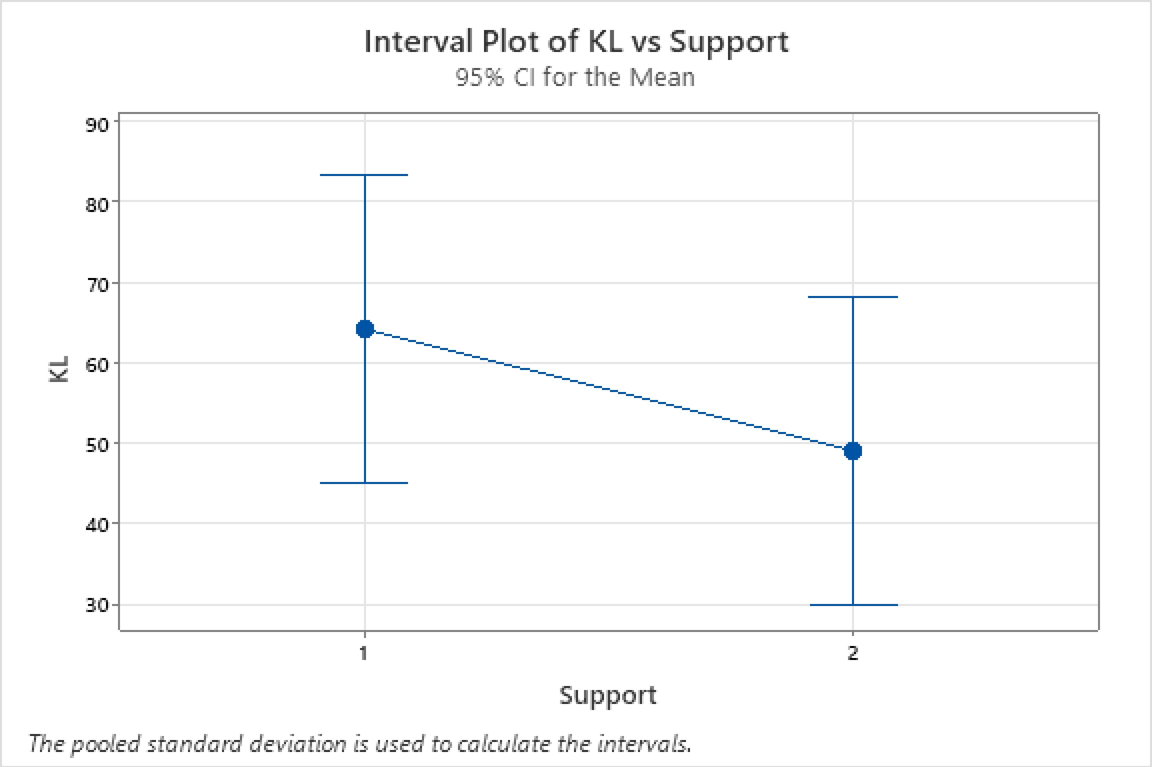
Interval Plot of KL vs Support
95% CI for the Mean
KL
90
80
70
60
50
40
30
1
2
Support
The pooled standard deviation is used to calculate the intervals.

One-way ANOVA: 6M Acid KL versus Support

(1 = membrane, 2= resin)

Method

Null hypothesis	All means are equal
Alternative hypothesis	Not all means are equal
Significance level	α = 0.05

Equal variances were assumed for the analysis.

Factor Information

Factor	Levels	Values
Support	2	1, 2

Analysis of Variance

Source	DF	Adj SS	Adj MS	F-Value	P-Value
Support	1	955.6	955.56	13.15	0.022
Error	4	290.6	72.64		
Total	5	1246.1			

Model Summary

S	R-sq	R-sq(adj)	R-sq(pred)
8.52309	76.68%	70.85%	47.53%

Means

Support	N	Mean	StDev	95% CI
1	3	7.85	7.09	(-5.82, 21.51)
2	3	33.09	9.75	(19.42, 46.75)

Pooled StDev = 8.52309

Tukey Pairwise Comparisons

Grouping Information Using the Tukey Method and 95% Confidence

Support	N	Mean	Grouping	
2	3	33.09	A	
1	3	7.85		B

Means that do not share a letter are significantly different.

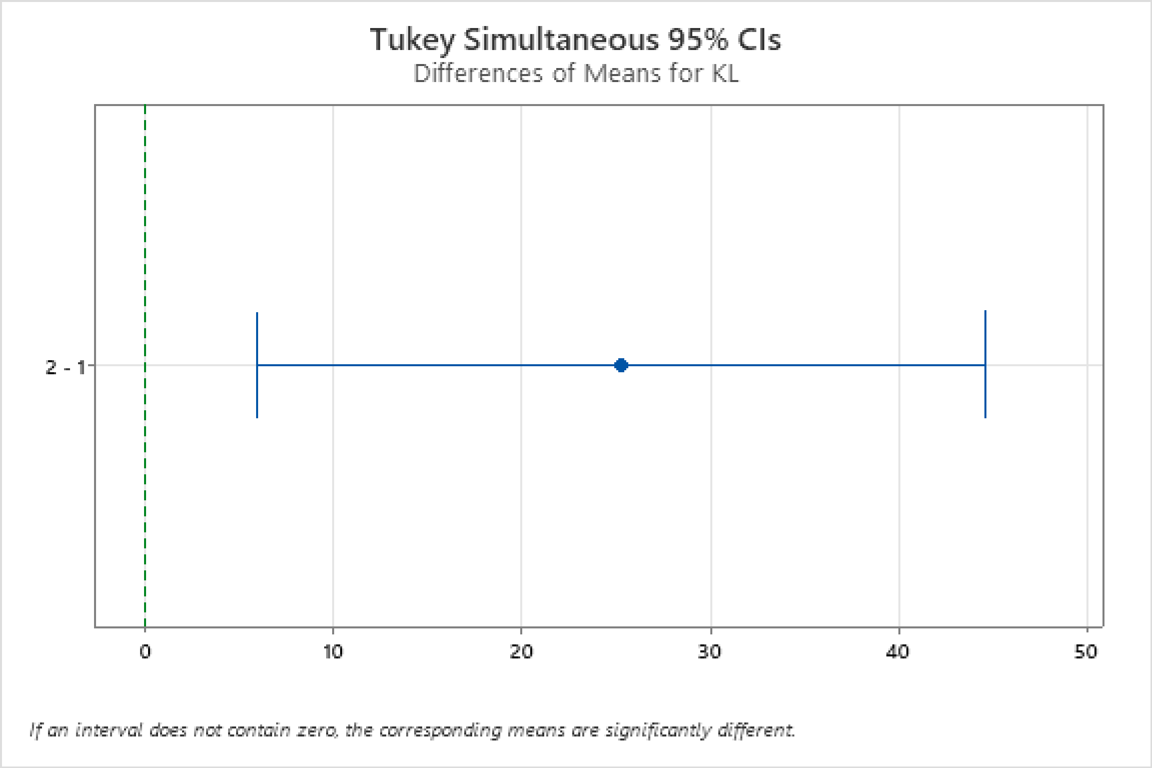
Tukey Simultaneous 95% CIs
Differences of Means for KL
2 - 1
0
10
20
30
40
50
If an interval does not contain zero, the corresponding means are significantly different.

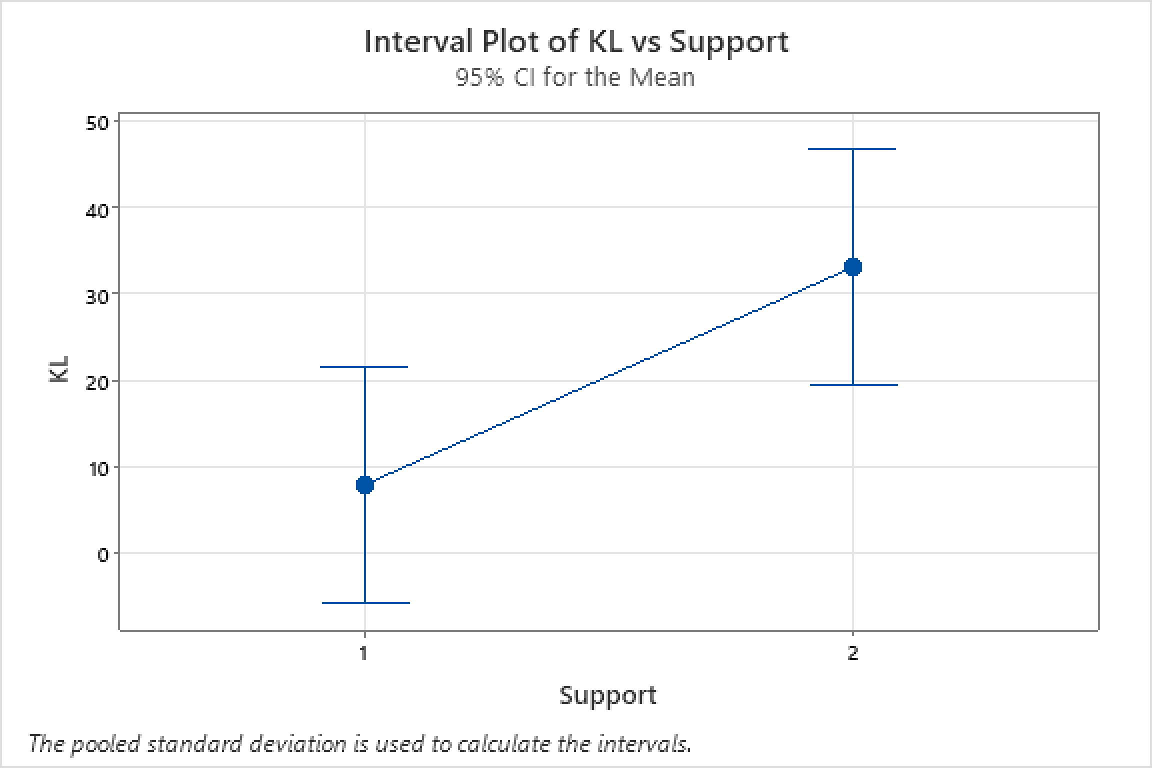
Interval Plot of KL vs Support
95% CI for the Mean
KL
50
40
30
20
10
0
1
2
Support
The pooled standard deviation is used to calculate the intervals.

One-way ANOVA: 10 M Acid KL versus Support

(1 = membrane, 2= resin)

Method

Null hypothesis	All means are equal
Alternative hypothesis	Not all means are equal
Significance level	α = 0.05

Equal variances were assumed for the analysis.

Factor Information

Factor	Levels	Values
Support	2	1, 2

Analysis of Variance

Source	DF	Adj SS	Adj MS	F-Value	P-Value
Support	1	543.46	543.459	94.78	0.001
Error	4	22.94	5.734		
Total	5	566.39			

Model Summary

S	R-sq	R-sq(adj)	R-sq(pred)
2.39453	95.95%	94.94%	90.89%

Means

Support	N	Mean	StDev	95% CI
1	3	6.90	3.04	(3.06, 10.74)
2	3	25.931	1.488	(22.093, 29.769)

Pooled StDev = 2.39453

Tukey Pairwise Comparisons

Grouping Information Using the Tukey Method and 95% Confidence

Support	N	Mean	Grouping	
2	3	25.931	A	
1	3	6.90		B

Means that do not share a letter are significantly different.

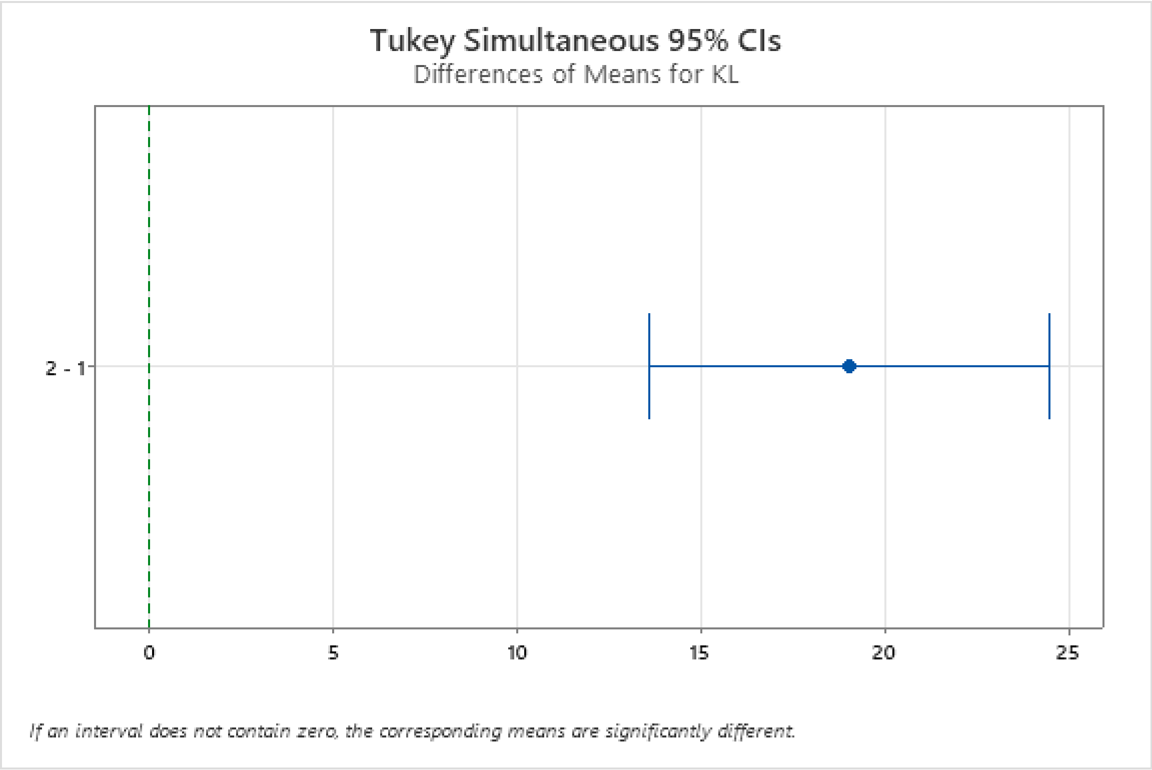
Tukey Simultaneous 95% CIs
Differences of Means for KL
2 - 1
0
5
10
15
20
25
If an interval does not contain zero, the corresponding means are significantly different.

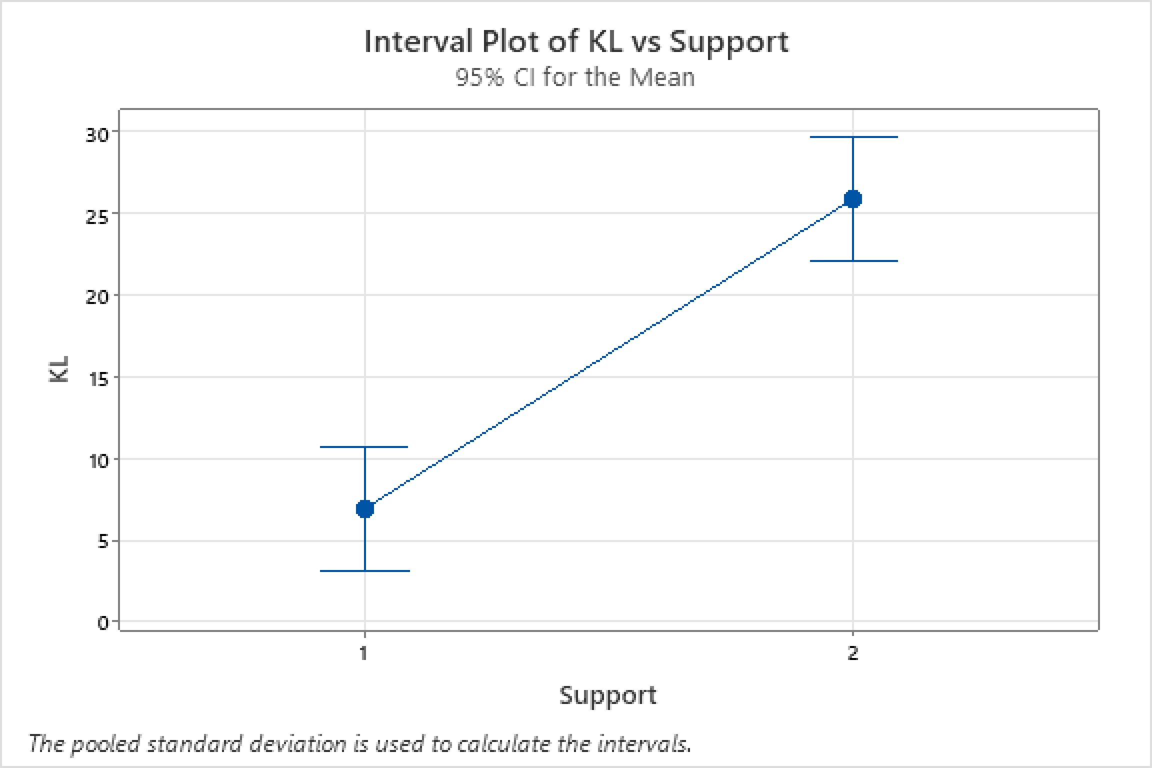
Interval Plot of KL vs Support
95% CI for the Mean
KL
30
25
20
15
10
5
0
1
2
Support
The pooled standard deviation is used to calculate the intervals.

One-way ANOVA: 4 M Acid Qmax versus Support

(1 = membrane, 2= resin)

Method

Null hypothesis	All means are equal
Alternative hypothesis	Not all means are equal
Significance level	α = 0.05

Equal variances were assumed for the analysis.

Factor Information

Factor	Levels	Values
Support	2	1, 2

Analysis of Variance

Source	DF	Adj SS	Adj MS	F-Value	P-Value
Support	1	0.026812	0.026812	1556.83	0.000
Error	4	0.000069	0.000017		
Total	5	0.026881			

Model Summary

S	R-sq	R-sq(adj)	R-sq(pred)
0.0041500	99.74%	99.68%	99.42%

Means

Support	N	Mean	StDev	95% CI
1	3	0.001870	0.000426	(-0.004782, 0.008522)
2	3	0.13557	0.00585	(0.12891, 0.14222)

Pooled StDev = 0.00414997

Tukey Pairwise Comparisons

Grouping Information Using the Tukey Method and 95% Confidence

Support	N	Mean	Grouping	
2	3	0.13557	A	
1	3	0.001870		B

Means that do not share a letter are significantly different.

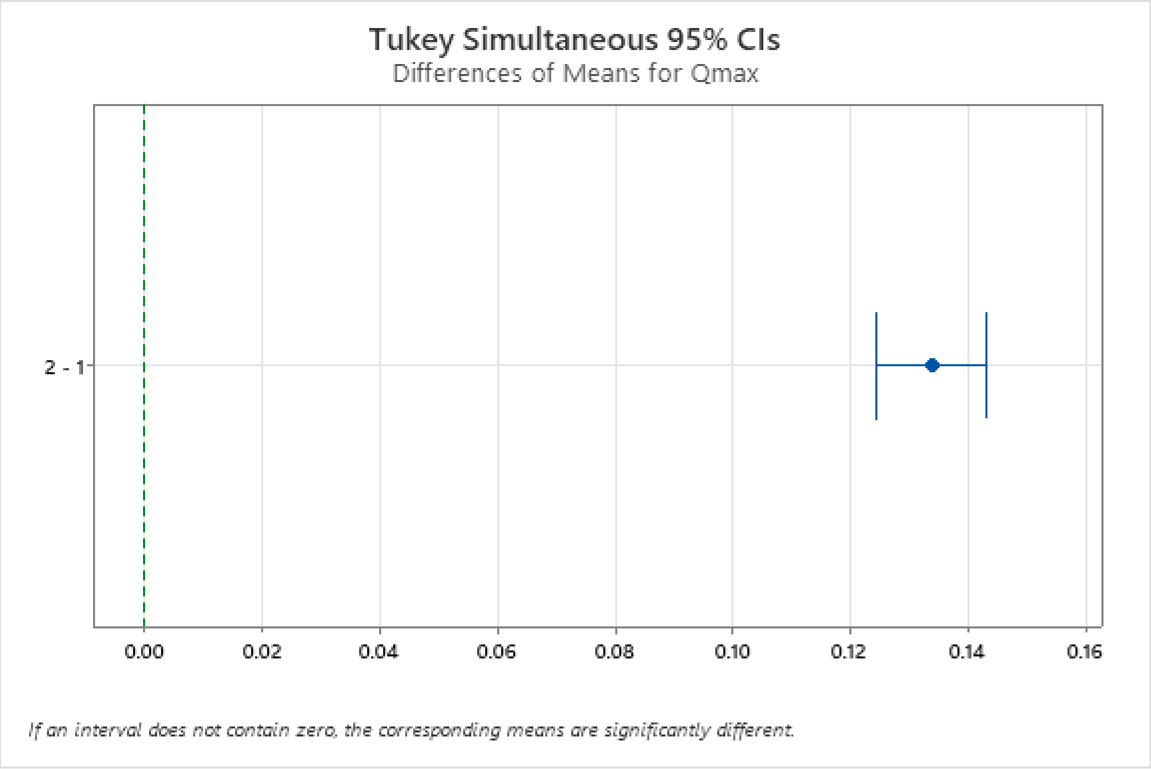
Tukey Simultaneous 95% CIs
Differences of Means for Qmax
2 - 1
0.00
0.02
0.04
0.06
0.08
0.10
0.12
0.14
0.16
If an interval does not contain zero, the corresponding means are significantly different.

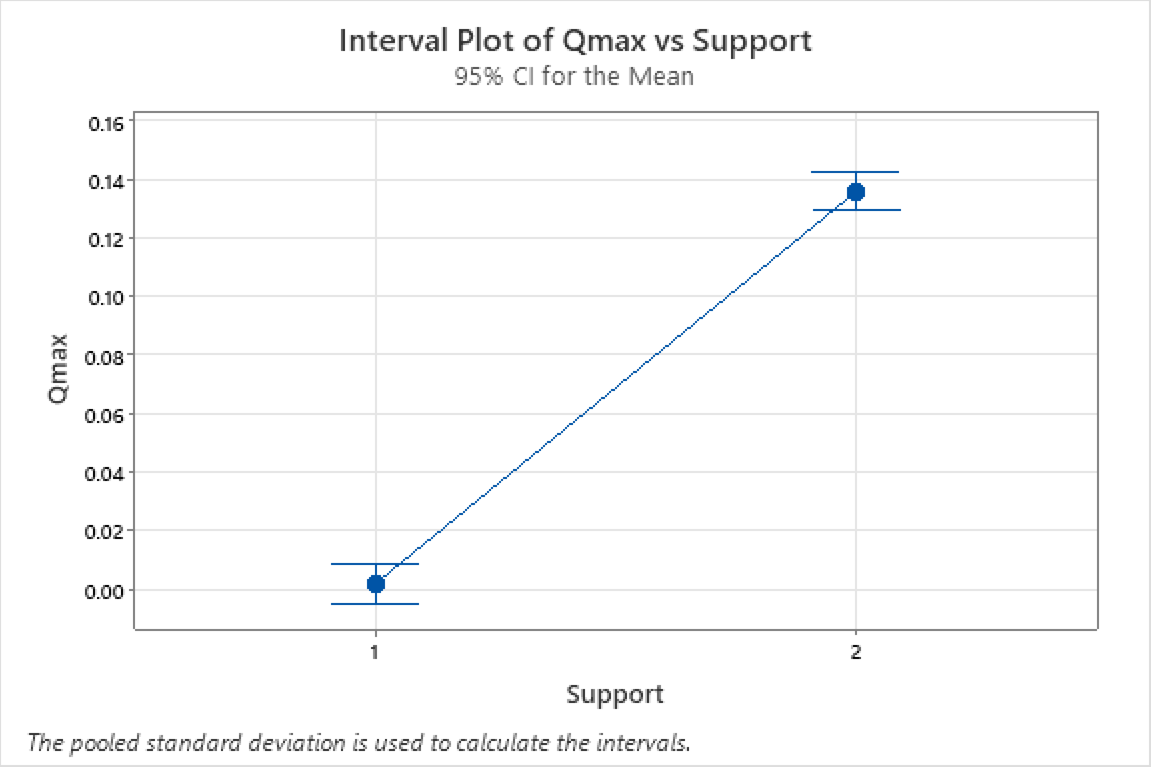
Interval Plot of Qmax vs Support
95% CI for the Mean
Qmax
0.16
0.14
0.12
0.10
0.08
0.06
0.04
0.02
0.00
1
2
Support
The pooled standard deviation is used to calculate the intervals.

One-way ANOVA: 6 M Acid Qmax versus Support

(1 = membrane, 2= resin)

Method

Null hypothesis	All means are equal
Alternative hypothesis	Not all means are equal
Significance level	α = 0.05

Equal variances were assumed for the analysis.

Factor Information

Factor	Levels	Values
Support	2	1, 2

Analysis of Variance

Source	DF	Adj SS	Adj MS	F-Value	P-Value
Support	1	0.021338	0.021338	387.90	0.000
Error	4	0.000220	0.000055		
Total	5	0.021558			

Model Summary

S	R-sq	R-sq(adj)	R-sq(pred)
0.0074169	98.98%	98.72%	97.70%

Means

Support	N	Mean	StDev	95% CI
1	3	0.00306	0.00216	(-0.00883, 0.01495)
2	3	0.12233	0.01026	(0.11044, 0.13422)

Pooled StDev = 0.00741685

Tukey Pairwise Comparisons

Grouping Information Using the Tukey Method and 95% Confidence

Support	N	Mean	Grouping	
2	3	0.12233	A	
1	3	0.00306		B

Means that do not share a letter are significantly different.

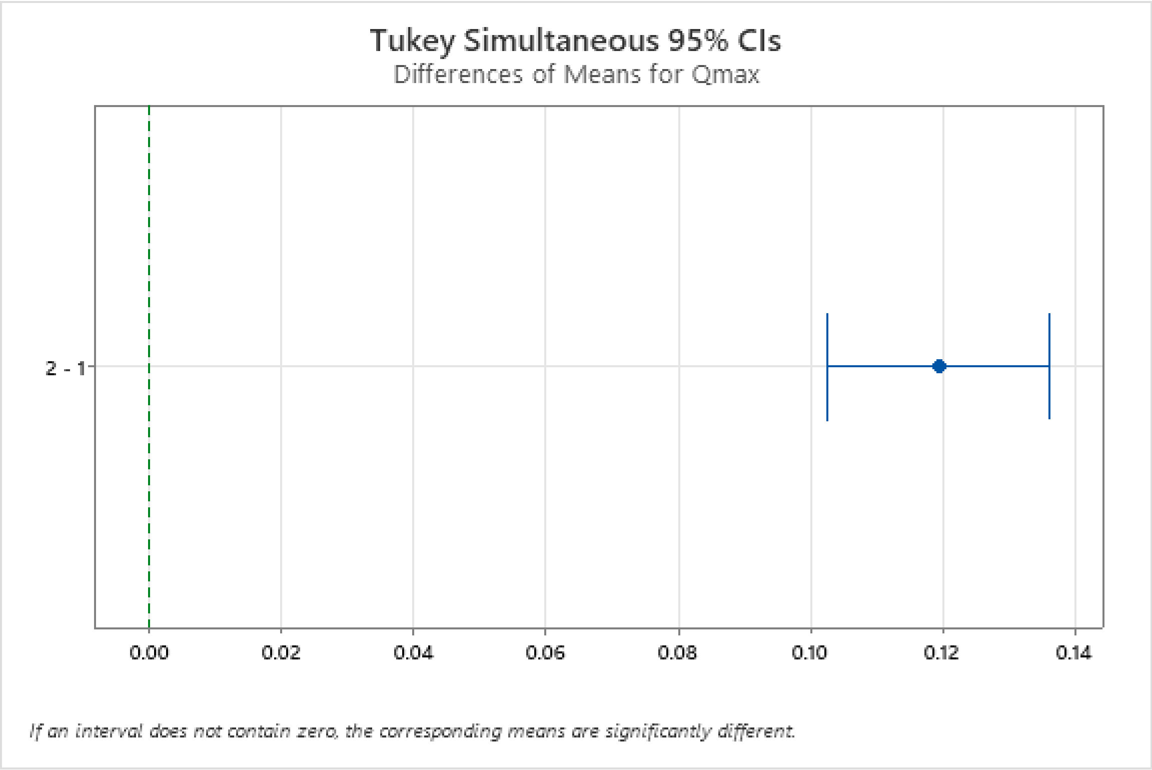
Tukey Simultaneous 95% CIs
Differences of Means for Qmax
2 - 1
0.00
0.02
0.04
0.06
0.08
0.10
0.12
0.14
If an interval does not contain zero, the corresponding means are significantly different.

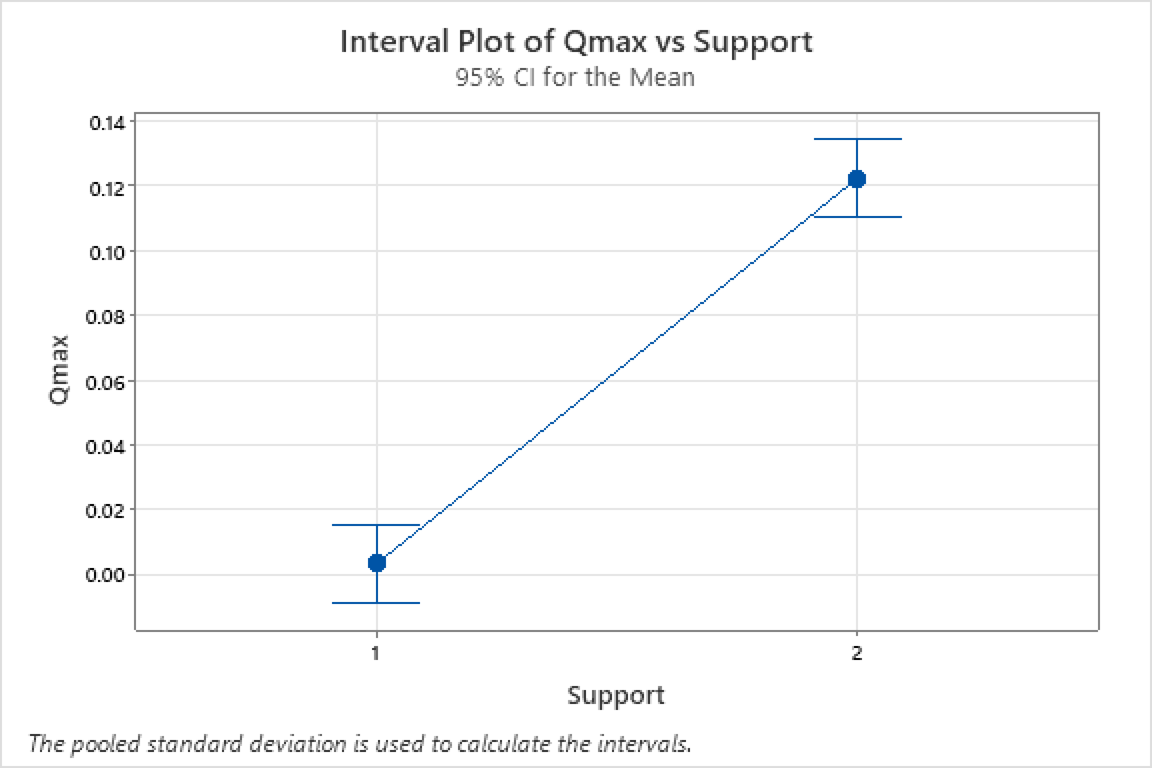
Interval Plot of Qmax vs Support
95% CI for the Mean
Qmax
0.14
0.12
0.10
0.08
0.06
0.04
0.02
0.00
1
2
Support
The pooled standard deviation is used to calculate the intervals.

One-way ANOVA: 10 M Acid Qmax versus Support

(1 = membrane, 2= resin)

Method

Null hypothesis	All means are equal
Alternative hypothesis	Not all means are equal
Significance level	α = 0.05

Equal variances were assumed for the analysis.

Factor Information

Factor	Levels	Values
Support	2	1, 2

Analysis of Variance

Source	DF	Adj SS	Adj MS	F-Value	P-Value
Support	1	0.007877	0.007877	715.39	0.000
Error	4	0.000044	0.000011		
Total	5	0.007921			

Model Summary

S	R-sq	R-sq(adj)	R-sq(pred)
0.0033183	99.44%	99.30%	98.75%

Means

Support	N	Mean	StDev	95% CI
1	3	0.013867	0.001720	(0.008548, 0.019186)
2	3	0.08633	0.00437	(0.08101, 0.09165)

Pooled StDev = 0.00331827

Tukey Pairwise Comparisons

Grouping Information Using the Tukey Method and 95% Confidence

Support	N	Mean	Grouping	
2	3	0.08633	A	
1	3	0.013867		B

Means that do not share a letter are significantly different.

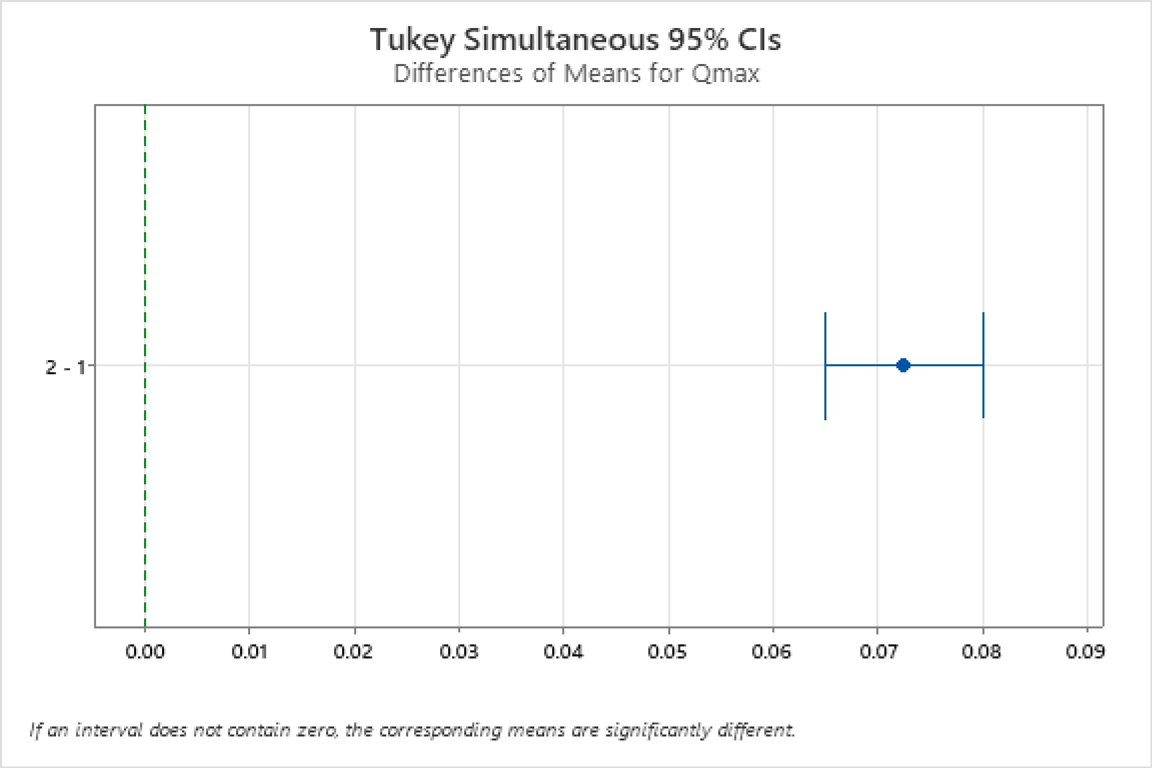
Tukey Simultaneous 95% CIs
Differences of Means for Qmax
2 - 1
0.00
0.01
0.02
0.03
0.04
0.05
0.06
0.07
0.08
0.09
If an interval does not contain zero, the corresponding means are significantly different.

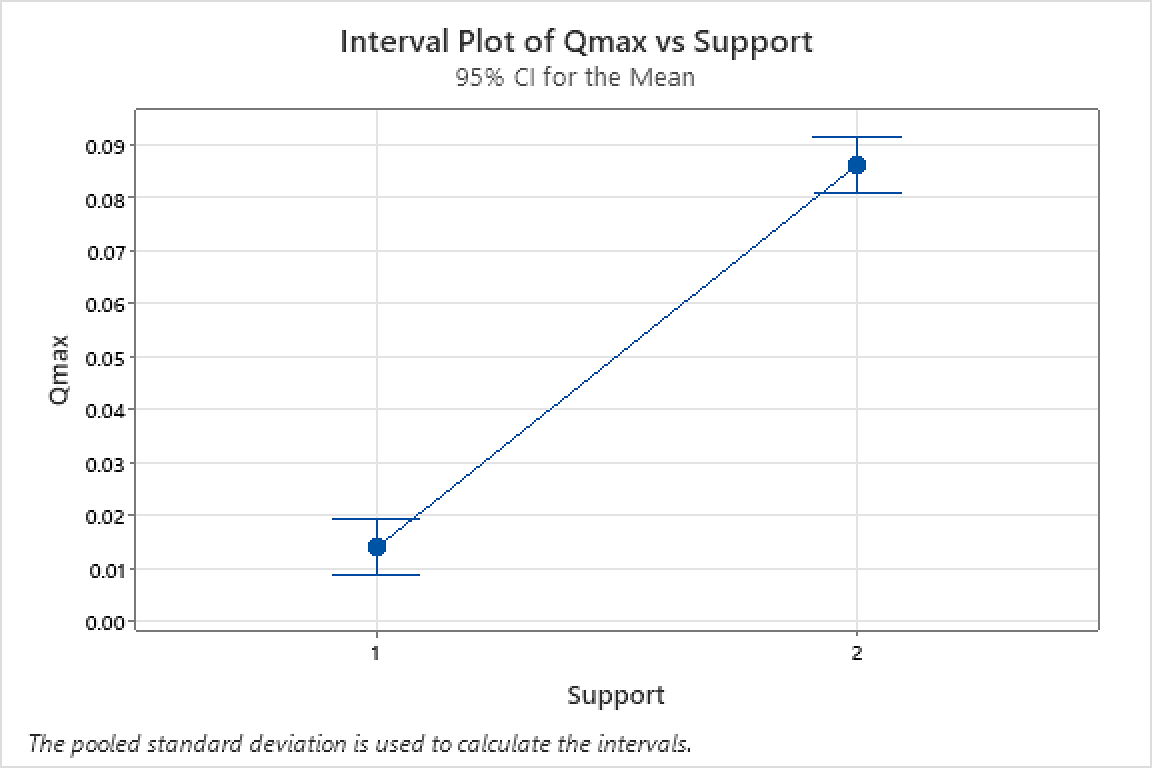
Interval Plot of Qmax vs Support
95% CI for the Mean
Qmax
0.09
0.08
0.07
0.06
0.05
0.04
0.03
0.02
0.01
0.00
1
2
Support
The pooled standard deviation is used to calculate the intervals.

Calculations to Support the Selectivity Discussion

The estimated distribution coefficient for La(III) on the THDGA membranes was modeled to match the ^{225}Ac adsorption experiments performed at BNL. In those experiments, the mass of membranes was 1.25 mg, the volume was 1 mL, and the activity concentration was 12 microCi/mL or 0.88 pmol/mL. The distribution coefficient for La(III) was calculated in two ways: 1) using the Langmuir equation to calculate the K_d for the given adsorbent mass, volume, and initial activity and 2) using the linear adsorption equation to calculate the K_d for the same adsorbent mass, volume, and initial activity. The results of the K_d calculations using both isotherm models are shown below, **Table S5.**

Table S5. Modeled distribution coefficients for La(III) at the same conditions as the BNL adsorption experiments. These distribution coefficients are later used to estimate the selectivity of the membranes for La/^{225}Ac in equimolar mixtures.

**Acid**	**K_d calculated by Langmuir Isotherm** (mL/g)	**K_d calculated by Linear** (mL/g)
4 M HNO_3	115	39.4
6 M HNO_3	14	33.7
10 M HNO_3	90	68.1

Table S6. Measured distribution coefficients for Ac-225 TODGA membranes.

**Acid**	**K_d experimentally measured** (mL/g)
4 M HNO_3	32.3 ± 2.7
6 M HNO_3	10.8 ± 1.9
10 M HNO_3	1.2 ± 3.9

The estimated selectivity of the membranes was calculated in two ways: 1) using experimental Ac-225 data and the K_d modeled by the Langmuir isotherm and 2) using experimental ^{225}Ac data and the K_d modeled by the Linear isotherm.

Table S7. Selectivity of the THDGA membranes calculated using the K_d calculated from the Langmuir isotherm fit and the linear isotherm fit.

**Acid**	**Selectivity ($K_{d,La}/K_{d,Ac}$) via Langmuir Isotherm**	**Selectivity ($K_{d,La}/K_{d,Ac}$) via Linear Isotherm**
4 M HNO_3	3.6	1.2
6 M HNO_3	1.3	3.1
10 M HNO_3	73.0	56.8

To compare the selectivity of the THDGA membrane to the TODGA resins, we used k' values from the literature which are summarized in **Table S8** for 4 M and 6 M nitric acid**.** All data was digitized from published works using the "Digitizer" tool in Origin 2023b (version 10.0.5.157) software. To compare

with TODGA resins at 10 M nitric acid, it was necessary to use experimental data from this work, shown in **Table S4** and digitized data from Radchenko et al., **Table S9.**

Table S8. Digitized k' data for La and ^{225}Ac adsorption on TODGA resins from multiple sources.

HNO_3 (M)	k' Ac-225[a]	k' Ac-225[b]	k' La[a]	k' La[c]
10	--	--	--	2300
8	123	72	27986	
7	--	205		
6	764	--	18666	
5	--	736		
4	1406		9347	
3	--	1110	--	
2	2290	--	3122	
1	1327	543	1070	
0.5	598	--	325	
0.25	75	--	112	
0.05	--	13	--	
0.01	Not reported	8	--	

[a] Horwitz, E. P.; McAlister, D. R.; Bond, A. H.; Barrans, R. E. Novel Extraction of Chromatographic Resins Based on Tetraalkyldiglycolamides: Characterization and Potential Applications. *Solvent Extr. Ion Exch.* **2005**, *23* (3), 319–344. https://doi.org/10.1081/sei-200049898.

[b] Aliev, R. A.; Ermolaev, S. V.; Vasiliev, A. N.; Ostapenko, V. S.; Lapshina, E. V.; Zhuikov, B. L.; Zakharov, N. V.; Pozdeev, V. V.; Kokhanyuk, V. M.; Myasoedov, B. F.; Kalmykov, S. N. Isolation of Medicine-Applicable Actinium-225 from Thorium Targets Irradiated by Medium-Energy Protons. *Solvent Extr. Ion Exch.* **2014**, *32* (5), 468–477. https://doi.org/10.1080/07366299.2014.896582

[c] Pourmand, A.; Dauphas, N. Distribution Coefficients of 60 Elements on TODGA Resin: Application to Ca, Lu, Hf, U and Th Isotope Geochemistry. *Talanta* **2010**, *81* (3), 741–753. https://doi.org/10.1016/j.talanta.2010.01.008

Table S9. Digitized K_d for ^{225}Ac from Radchenko et al.[d]

HNO_3 (M)	$K_{d, Ac-225}$ on DGA Normal (mL/g)	$K_{d, Ac-225}$ on DGA Branched (mL/g)
0.15	75	4.5
1	204	168
4	815	212
6	138	328
8	92	122
10	67	78
12	30	56

[d] Radchenko, V.; Engle, J. W.; Wilson, J. J.; Maassen, J. R.; Nortier, F. M.; Taylor, W. A.; Birnbaum, E. R.; Hudston, L. A.; John, K. D.; Fassbender, M. E. Application of Ion Exchange and Extraction Chromatography to the Separation of Actinium from Proton-Irradiated Thorium Metal for Analytical Purposes. *J. Chromatogr. A* **2015**, *1380*, 55–63. https://doi.org/10.1016/j.chroma.2014.12.045.
